# Electron-deficient two-dimensional poly(arylene vinylene) covalent organic frameworks: efficient synthesis and host–guest interaction†

**DOI:** 10.1039/d4sc06903j

**Published:** 2025-02-03

**Authors:** Albrecht L. Waentig, Xiaodong Li, Meng Zhao, Sattwick Haldar, Philomene Koko, Silvia Paasch, Alina Mueller, Karen M. Garcia Alvarez, Florian Auras, Eike Brunner, Andreas Schneemann, Jia-Qi Huang, Stefan Kaskel, Mingchao Wang, Xinliang Feng

**Affiliations:** a Center for Advancing Electronics Dresden (CFAED) and Faculty of Chemistry and Food Chemistry, Technische Universität Dresden Mommsenstrasse 4 01069 Dresden Germany stefan.kaskel@tu-dresden.de xinliang.feng@tu-dresden.de; b Max Planck Institute of Microstructure Physics Weinberg 2 06120 Halle Germany; c School of Materials Science and Engineering, Beijing Institute of Technology Beijing 100081 China; d Advanced Research Institute of Multidisciplinary Science, Beijing Institute of Technology Beijing 100081 China; e Fraunhofer Institute for Material and Beam Technology (IWS) Winterbergstraße 28 01277 Dresden Germany; f School of Advanced Materials, Peking University, Shenzhen Graduate School Shenzhen 518055 China mingchao.wang@pku.edu.cn

## Abstract

Crystalline and porous 2D poly(arylene vinylene)s (2D PAVs), *i.e.* vinylene-linked 2D conjugated covalent organic frameworks, represent promising materials for electronic and electrochemical applications. Chemically robust 2D PAVs with strong electron affinity are highly desirable for effective host–guest charge transfer to achieve enhanced device performance. Herein, we report the efficient synthesis and host–guest interaction of two novel 2D PAVs incorporating electron-deficient bipyrazine units with a N-free 2D PAV as a reference. They are crystalline and chemically robust. Various spectroscopies coupled with theoretical calculations indicate that the abundant N sites boost the electron affinity of 2D PAVs. We test their efficiency in hosting guest sulfur species and find that the electron-deficient materials help to physically confine and stabilize sulfur/polysulfide (*e.g.*, Li_2_S_6_) molecules with facilitated intermolecular charge transfer in the porous channels. As a result, using sulfur encapsulated by 2D PAVs as electrode materials, we achieve high specific capacities with excellent capacity retention after 200 charge–discharge cycles for Li–sulfur batteries.

## Abstract 2

Two electron-deficient crystalline 2D poly(arylene vinylene)s are developed to stabilize sulfur/polysulfide molecules with facilitated host–guest charge transfer, which contributes to enhanced lithium–sulfur battery performance.

## Graphical abstract

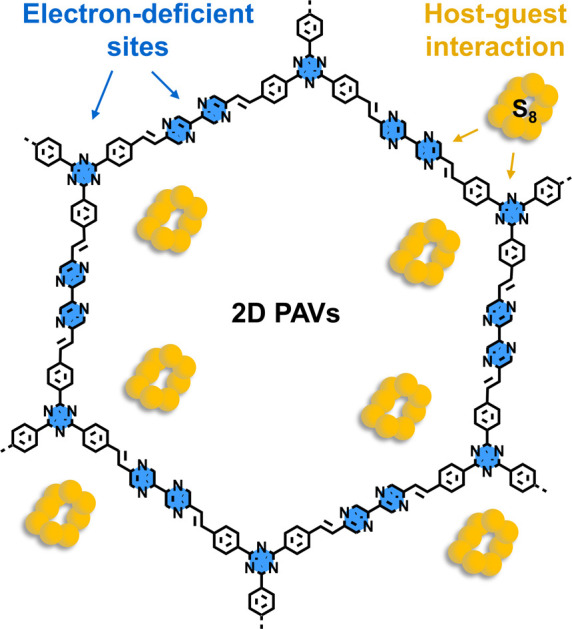

## Introduction

Two-dimensional conjugated covalent organic frameworks (2D c-COFs) belong to a class of layer-stacked and crystalline 2D conjugated polymers that are linked by conjugated linkages such as C=N bonds (including imines,^1–3^ pyrazines,^4^ and triazines^5^), vinylenes (or sp^2^-carbon),^6,7^ thiazoles,^8^ (2-phenyl)imidazoles,^9^ ladder-type imidazoles,^10^*etc.* These materials exhibit extended in-plane π-conjugation^10^ and out-of-plane electronic couplings.^4^ Benefiting from their well-defined and tailorable porous structures, intrinsic charge carrier mobilities (up to 1000 cm^2^ V ^−1^ s^−1^),^10^ and abundant active sites, they have attracted considerable attention in applications including (opto)electronics,^11,12^ electro-/photo-/photoelectrochemical catalysis,^13–16^ and electrochemical energy storage (*e.g.*, supercapacitors and metal-ion batteries).^17–20^ In particular, vinylene-linked 2D c-COFs, *i.e.*, 2D poly(arylene vinylene)s (2D PAVs), developed *via* Knoevenagel,^6,7^ Horner–Wadsworth–Emmons (HWE),^21^ Wittig,^22^ aldol-type,^23–26^ or Claisen–Schmidt^27^ polycondensation reactions, have demonstrated excellent π-conjugation and chemical/thermal stabilities, which are superior to those of C=N-linked 2D c-COFs and highlight the great potential of these materials in functional applications.

To date, considerable efforts have been dedicated to engineering the 2D PAV backbone with electron-rich molecular units such as pyrene,^7^ porphyrin,^28^ benzotrithiophene^29,30^ or thienyl-benzodithiophene^31^ with the aim of enhancing the 2D π-conjugation towards polymer semiconductors with narrow bandgaps and boosted charge-carrier mobilities.^10,31^ In comparison, electron-deficient materials possess the low-lying lowest unoccupied molecular orbital (LUMO) and high electronegativity, which can facilitate charge carrier separation and intermolecular charge transfer to enhance the performance for a wide range of applications, such as n-type organic field-effect transistors^32^ and photocatalysis.^33^ Moreover, a porous and electron-deficient 2D PAV skeleton can serve as an effective host for electron-rich molecules with facilitated host–guest charge transfer, which could boost the performance of, for example, organic solar cells, and metal–sulfur/selenium batteries. Although a few electron-deficient molecular units, *e.g.*, pyrazine^21,22,34^ and triazine,^23,24,35^ have been sparsely incorporated into these materials, the development of electron-deficient 2D PAVs and their related applications have remained largely unexplored.

Herein, we report the efficient synthesis and host–guest interaction of two novel 2D PAVs incorporating electron-deficient bipyrazine (BPZ) units (termed 2DPAV-TPB-BPZ and 2DPAV-TPT-BPZ, where TPB = triphenylbenzene and TPT = triphenyltriazine). They were synthesized from 5,5′-dimethyl-2,2′-bipyrazine (DMBP) and aldehyde monomers with different electron-deficiencies *via* an aldol-type 2D polycondensation under solvothermal conditions. A TPB/biphenyl (BP)-based electron-neutral 2D PAV (2DPAV-TPB-BP) was also studied as a reference. Various spectroscopies coupled with theoretical calculations indicate that the abundant N sites boost the electron affinity of 2D PAVs in the sequence of 2DPAV-TPT-BPZ > 2DPAV-TPB-BPZ > 2DPAV-TPB-BP. We tested their efficiency in hosting guest sulfur/polysulfide molecules. Remarkably, their robust polymer backbones retain high crystallinity after the sulfurization, which is essential to elucidate structure–property relationships. We found that the electron deficiency of 2D PAVs helps to physically confine and stabilize guest sulfur/polysulfide (*e.g.*, Li_2_S_6_) molecules in their porous channels with facilitated intermolecular interactions. As a proof-of-concept application, we fabricated electrodes from sulfur encapsulated by 2D PAVs, which can deliver a high specific capacity and show excellent capacity retention of 80% after 200 charge–discharge cycles for the Li-–sulfur batteries.

## Results and discussion

### Design and synthesis of electron-deficient 2D PAVs

**Fig. 1:**
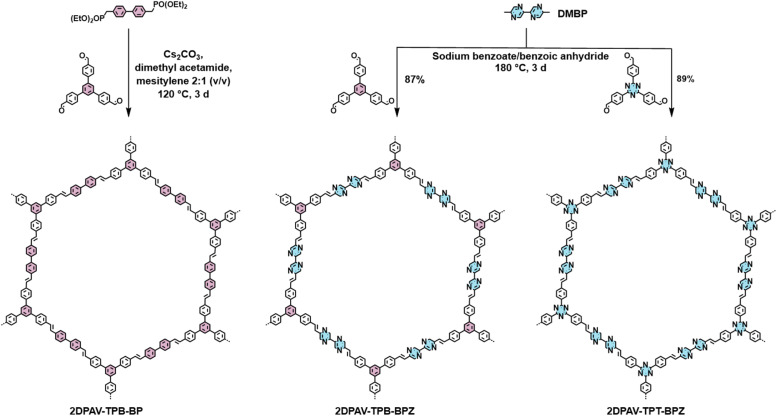
Synthetic scheme of 2D PAVs.

To tune the energy levels and control the electron density in 2D PAVs, we selected TPB- or TPT-based 2D PAVs with a honeycomb lattice (2DPAV-TPB-BP, 2DPAV-TPB-BPZ, and 2DPAV-TPT-BPZ; see the chemical structures in Fig. 1) and designed 11 pyrazine/triazine-based vinylene-linked model compounds with gradually increasing N/C atomic ratio from 0 to 0.47 (see details in Fig. S1†). We calculated the frontier-orbital energy levels of the model compounds using the density functional theory (DFT) method. Due to the cross-conjugation in the *C*_3_-symmetric molecules, they exhibit similar HOMO (highest occupied molecular orbital)–LUMO gaps of *ca.* 3.3. eV, while their HOMO/LUMO energy levels simultaneously decrease following a linear relationship with the increase of N/C atomic ratios (see details in Fig. S2 and S3†). This implies a great feasibility of enhancing the electron deficiency, *i.e.* lowering the LUMO level by increasing the N density in 2D PAVs. We further calculated the charge density distribution and electrostatic potentials of 2D PAVs. In contrast to 2DPAV-TPB-BP, the distribution images indicate strong polarization of total electron density in the BPZ-bridged 2D PAVs (Fig. S4–6†).

Towards the synthesis of 2D PAVs, the DMBP monomer was first synthesized by a Zn-catalyzed Negishi-type homo-coupling of 2-bromo-5-methylpyrazine (see details in the ESI†). 2DPAV-TPB-BPZ and 2DPAV-TPT-BPZ were then synthesized *via* aldol-type 2D polycondensation between DMBP and 1,3,5-tris(4-formylphenyl)benzene or 4,4′,4′′-(1,3,5-triazine-2,4,6-triyl)tris[benzaldehyde] using sodium benzoate and benzoic anhydride as a mixed catalyst at 180 °C for 3 days (Fig. 1, the optimized reaction conditions are shown in Table S1†). 2DPAV-TPB-BP as the reference sample was synthesized using a previously reported HWE polycondensation method.^21^ It is worth noting that, although benzoic acid and/or benzoic anhydride are standard catalysts for aldol-type 2D polycondensation, the presence of sodium benzoate here is essential to slow down the condensation (see the *in situ* model reactions in Fig. S7 and S8†) towards enhanced reaction reversibility and thus achieve crystalline electron-deficient 2D PAVs.

**Fig. 2:**
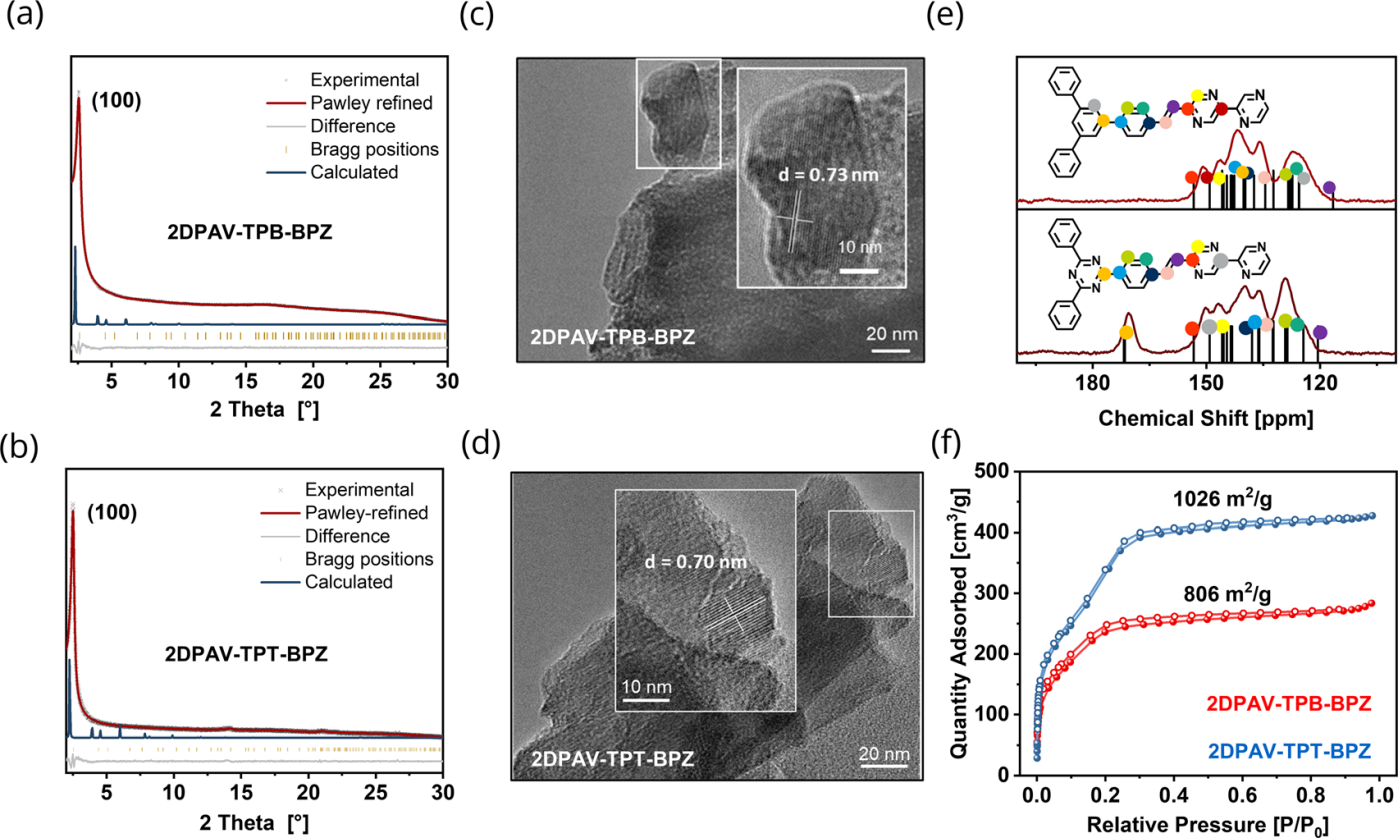
Characterization of 2DPAV-TPB-BPZ and 2DPAV-TPT-BPZ. (a and b) Experimental and calculated PXRD patterns, respectively. (c and d) TEM images of 2DPAV-TPB-BPZ and 2DPAV-TPT-BPZ, respectively. (e) ^13^C CP NMR spectra of 2DPAV-TPB-BPZ (up) and 2DPAV-TPT-BPZ (down). Predicted spectra are displayed in black with the corresponding signal assignment. (f) Nitrogen physisorption isotherms measured at 77 K. Filled and closed symbols represent adsorption and desorption, respectively.

The formation of crystalline 2D PAVs was confirmed by powder X-ray diffraction (PXRD). Both 2DPAV-TPB-BPZ and 2DPAV-TPT-BPZ show sharp reflections at 2*θ* = *ca.* 2.5° corresponding to their (100) crystallographic planes (Fig. 2a and b). The experimental PXRD patterns match well with their simulated structures in an AA interlayer arrangement (see the Pawley refinement data in Fig. S9 and S10†). Scanning electron microscopy (SEM) images reveal rod-like morphology of the synthesized 2D PAVs (Fig. S11 and S12†). High-resolution transmission electron microscopy (TEM) images indicate the layer-stacked nature of 2DPAV-TPB-BPZ (Fig. 2c) and 2DPAV-TPT-BPZ (Fig. 2d) with bilayer distances of 7.3 and 7.0 Å, *i.e.* interlayer distances of ∼3.7 and ∼3.5 Å, respectively. The larger layer spacing in the former stems from its more twisted TPB core than the TPT core in the latter.

The chemical identity of the developed 2D PAVs was further characterized by nuclear magnetic resonance (NMR) and Fourier-transform infrared (FT-IR) spectroscopy. The solid-state ^13^C cross polarization (CP) NMR spectra display the signal of vinylene C atoms at *ca.* 123 ppm and other C signals related to the phenyl and BPZ units, while 2DPAV-TPT-BPZ presents an additional signal at 170 ppm for the triazine moieties (Fig. 2e, see the predicted NMR spectra in Fig. S13†). No obvious aldehyde C peak exists above 170 ppm, indicating the successful polycondensation of the monomers. The formation of vinylene linkages is further evident from the appearance of bands at 3000 cm^−1^ (*ν*(C–H)) in the FT-IR spectra and the disappearance of the (*ν*(C–H)) vibrational bands related to aldehyde groups at 2640–2860 cm^−1^ and methyl moieties of the monomers (Fig. S14†). A similar phenomenon is observed in 2DPAV-TPB-BP, which confirms the vinylene-linkage formation (Fig. S15†). Moreover, strong bands at *ca.* 1470 cm^−1^ (*ν*(C=C)) and *ca.* 1160 cm^−1^ (*ν*(C=N)) confirm the presence of phenyl and pyrazine units in the 2D PAVs.

The permanent porosity of the synthesized 2D PAVs was examined by N_2_ physisorption experiments at 77 K. 2DPAV-TPB-BPZ and 2DPAV-TPT-BPZ show features of type I and IV isotherms, indicating a combination of micro- and mesopores within the structure. The total N_2_ uptake values are 270 and 420 cm^3^ g^−1^, and Brunauer–Emmett–Teller (BET) surface areas are calculated to be 800 and 1026 m^2^ g^−1^, respectively (Fig. 2f). The pore sizes determined by the nonlocal DFT method are 3.82 and 4.02 nm, respectively (Fig. S16†), which are consistent with the refined chemical structures. Thermogravimetric analyses confirm the thermal stability of both samples displaying no obvious weight loss up to 500 °C (Fig. S17†).

To provide insight into their optical properties, we dispersed the powder-based samples in 2-propanol and measured their UV-visible absorption and fluorescence spectra (Fig. S18†). 2DPAV-TPB-BPZ and 2DPAV-TPT-BPZ present similar absorption edges at around 520 nm, which is slightly larger than that of 2DPAV-TPB-BP, corresponding to an almost identical optical band gap of *ca.* 2.5 eV, determined from Tauc plots (Fig. S19†). This is in agreement with the calculated results for the model compounds: tuning the N/C atomic ratio does not vary the HOMO–LUMO gap energy in the cross-conjugated arylene-vinylenes. Moreover, 2DPAV-TPB-BPZ and 2DPAV-TPT-BPZ exhibit pronounced fluorescence with an emission maximum at around 570 nm (Fig. S18†).

### Host–guest interaction between sulfur/sulfide and 2D PAVs

**Fig. 3:**
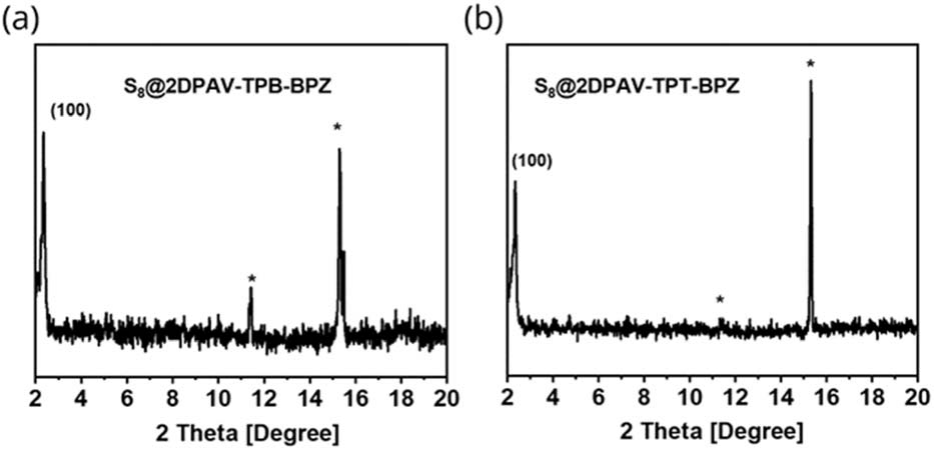
PXRD patterns of the sulfurized 2D PAVs with 86 wt% sulfur. (a) S_8_@2DPAV-TPB-BPZ. (b) S_8_@2DPAV-TPT-BPZ. Sulfur signals are marked by asterisks.

Given that electronegativity can largely influence the intermolecular interaction (*e.g.*, hydrogen bonding and electrostatic interaction) of organic compounds with guest molecules,^36^ we studied the physical confinement of elemental sulfur (*i.e.* S_8_) in these COF hosts. In this context, we prepared the 2D PAV–S_8_ composites, including S_8_@2DPAV-TPB-BP, S_8_@2DPAV-TPB-BPZ, and S_8_@2DPAV-TPT-BPZ, using typical sulfur-encapsulation conditions, *i.e. via* melt infiltration by heating a physical mixture of 2D PAV and S_8_ powders at 155 °C (ref. 37) for 6 h. Notably, the sulfurized samples still exhibit sharp PXRD signals corresponding to the 2D PAVs (Fig. 3), demonstrating the integrity of the robust, crystalline polymer networks after the sulfur-treatment, which is essential for elucidating an unambiguous structure–property relationship.

Taking S_8_@2DPAV-TPT-BPZ as an example, it exhibits a (100) peak at 2*θ* = *ca.* 2.5° with an identical FWHM value of *ca.* 0.40 to the pristine material. By contrast, both the chemical and physical sulfurization are associated with the amorphization of the reported COFs,^35,37–40^ most possibly due to the insufficient structural robustness. By modulating the weight ratio of 2D PAVs to S_8_, the sulfur content can be tuned from 60 to 86 wt% in the composites, which is estimated by gravimetric and thermogravimetric analyses (Fig. S17†) as well as elemental analysis (Table S2†). In comparison, we also conducted sulfurization of 2DPAV-TPT-BPZ at a much higher temperature of 350 °C to trigger covalent bond formation^40,41^ between sulfur and the polymer backbone. The obtained sample shows an amorphous feature (Fig. S20†), which further implies a physical process for the above-mentioned sulfurization at 155 °C.

**Fig. 4:**
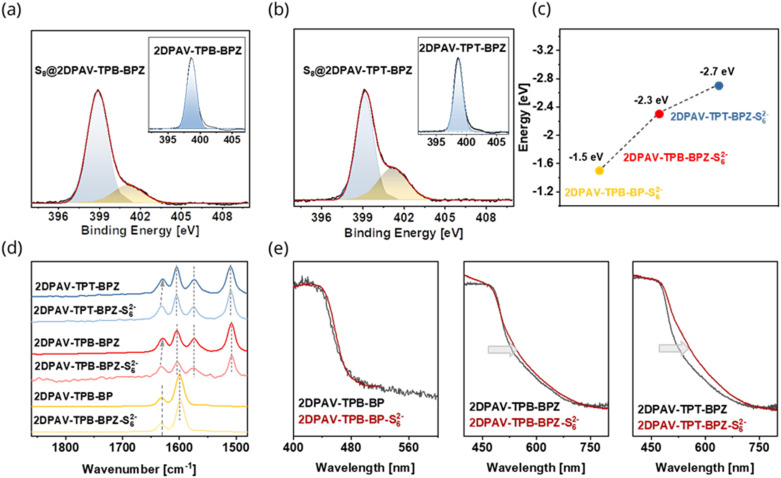
Intermolecular charge transfer between sulfur/polysulfide and 2D PAVs. (a and b) N 1s XPS spectra of 2D PAV–S_8_ composites. The insets show the N 1s XPS spectra of pristine 2D PAVs. (c) Adsorption energies of various 2D PAVs to S_6_^2−^ anions. The samples are named 2D PAV–S_6_^2−^. (d) Raman spectra of pristine 2D PAVs and the Li_2_S_6_ treated 2D PAVs. (e) Solid-state UV-visible absorption spectra of the pristine and Li_2_S_6_ treated 2D PAVs.

To confirm the strong interactions between 2D PAVs and sulfur/sulfide, we conducted X-ray photoelectron spectroscopy (XPS) analysis on the 2D PAV–S_8_ composites. The spectra suggest that the C 1s signals are insensitive to the host–guest interaction (Fig. S21†), while the N 1s spectra display an additional shoulder peak in the sulfur-confined 2D PAVs (Fig. 4a and b). Deconvolution of the N 1s signal generates peaks at 399.1 and 401.2 eV, which is attributed to the pristine N atoms in 2D PAVs and the partially charged N atoms (due to partial charge transfer between sulfur and 2D PAV), respectively. These results disclose that N atoms indeed function as active sites for the adsorption of S_8_ in 2D PAVs. The amounts of the emerging nitrogen species (N⋯S_8_) are calculated to be 13.8% and 23.5% for S_8_@2DPAV-TPB-BPZ and S_8_@2DPAV-TPT-BPZ, respectively, which suggests that the electron-deficient 2D PAV favors the host–guest interaction.

To elucidate the interaction of sulfur/polysulfides and 2D PAVs, we used DFT calculations to determine the adsorption energy of S_8_ (Fig. S22–S24†) or the S_6_^2−^ anion to the 2D PAVs (Fig. S25–S27†). S_6_^2−^ shows an adsorption energy of −2.7 eV to 2DPAV-TPT-BPZ (Fig. 4c and S25†), which is considerably higher than that of −0.19 eV for S_8_-adsorption (see the details of S_8_-adsorption in Fig. S22†). This indicates a significantly enhanced intermolecular charge-transfer behavior in the former. Moreover, 2DPAV-TPB-BPZ and 2DPAV-TPB-BP show inferior adsorption energies of −2.3 and −1.5 eV, respectively, for S_6_^2−^ (Fig. 4c, S26 and S27†).

We then prepared the 2D PAV–S_6_^2−^ composites by dropping a 0.1 M Li_2_S_6_ solution in 1 : 1 DOL/DME onto 2D PAV powders under an inert atmosphere and conducted solid-state Raman UV-visible spectroscopy measurements. Raman spectra show identical peaks for 2DPAV-TPB-BP before and after the Li_2_S_6_ treatment (Fig. 4d). While for 2DPAV-TPB-BPZ and 2DPAV-TPT-BPZ, the C=N vibrational band at 1629 cm^−1^ shifts to 1633 cm^−1^ in the 2D PAV–S_6_^2−^ composites due to the partial transfer from S_6_^2−^ to 2D PAVs (Fig. 4d, see the calculated Raman results and modes in Fig. S28†). In the UV-vis absorption spectrum, the composite of 2DPAV-TPT-BPZ and S_6_^2−^ presents a bathochromic shift of the absorption edge, compared to 2DPAV-TPT-BPZ, which clearly manifests the formation of a charge-transfer complex in the former (Fig. 4e). Such a phenomenon is less intense for the 2DPAV-TPB-BPZ composite and not observable for the 2DPAV-TPB-BP composite.

### 2D PAV–S_8_ composites for electrochemical energy storage

**Fig. 5:**
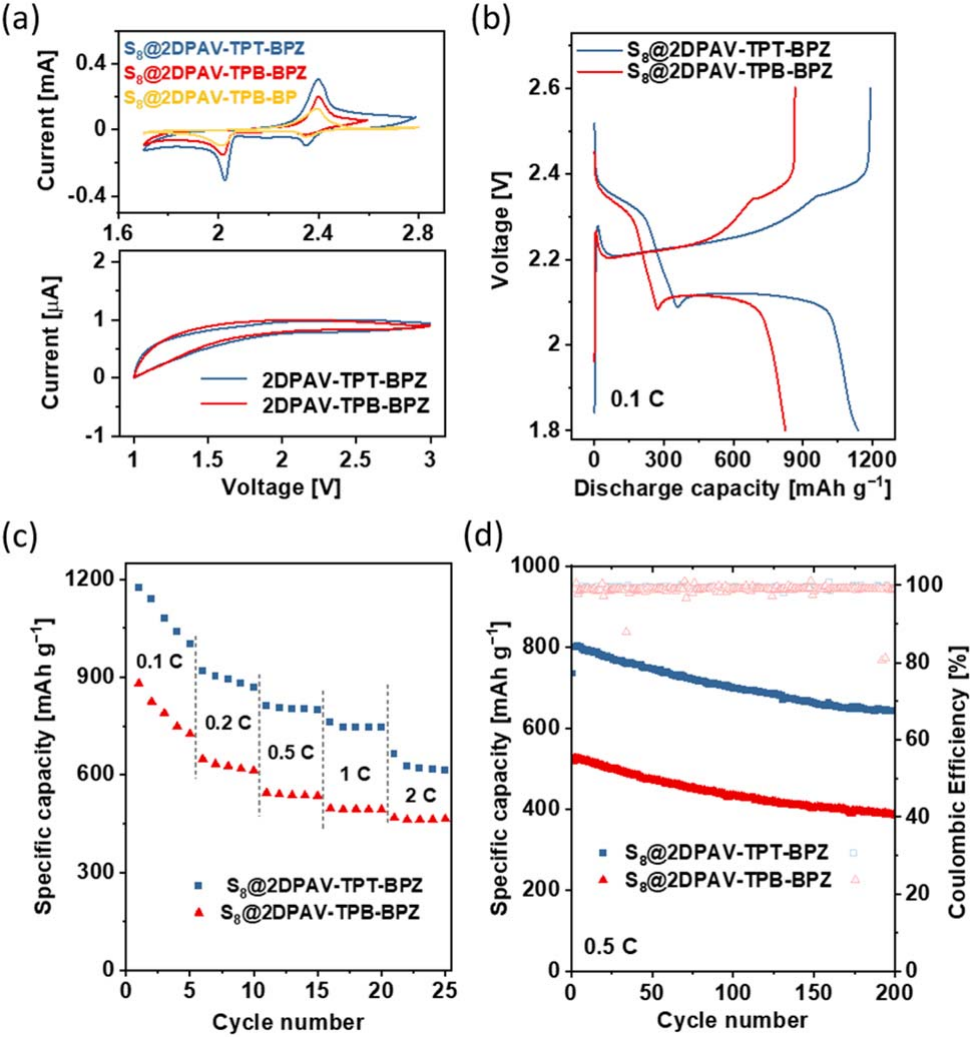
Electrochemical performance of 2D PAV–S_8_ composites as electrodes. (a) CV curves of various composites (60 wt% sulfur, top) and pristine 2D PAVs (down). (b) GCD curves of S_8_@2DPAV-TPB-BPZ and S_8_@2DPAV-TPT-BPZ with 86 wt% S_8_. (c) Rate performances. (d) Cycling performance. All the gravimetric discharge capacities are calculated based on sulfur.

A strong host–guest interaction between 2D PAV and sulfur species can confine the relatively mobile S_8_ and polysulfides within the porous hosts to achieve high capacity and alleviate the dissolution issue of polysulfide intermediates from sulfur electrodes in electrolytes. This makes such electron-deficient 2D PAVs promising hosts for metal–sulfur batteries. To this end, we fabricated cathodes from the 2D PAV–S_8_ (60 wt% sulfur) composites (see details in the ESI†) and tested their electrochemical performance. Cyclic voltammetry (CV) curves (Fig. 5a, top) show redox peaks related to the reduction of sulfur to sulfides (the detailed redox reaction of S_8_ is shown in Fig. S29†). The intensity of peak currents, which corresponds to the charge/discharge capacities of electrodes (see the galvanostatic charge discharge (GCD) curves in Fig. S30;† capacity in the range of 582–1300 mA h g_sulfur_^−1^), follows the same sequence as that of the electron affinity of 2D PAVs, *i.e.*, 2DPAV-TPT-BPZ > 2DPAV-TPB-BPZ > 2DPAV-TPB-BP. Under the same electrochemical conditions, the pristine 2D PAVs are not redox-active (Fig. 5a, down), which excludes the pseudocapacitive contribution of 2D PAVs and further highlights the importance of host–guest interaction in improving the battery performance.

We further investigated the electrochemical performance of 2D PAV–S_8_ composites with a higher sulfur loading (86 wt% S_8_). The CV and GCD curves are shown in Fig. S31† and 5b, respectively. At 0.1C, the S_8_@2DPAV-TPT-BPZ electrode with 86 wt% S_8_ still outputs a stable capacity of 1086 mA h g_sulfur_^−1^ (initial capacity of 1139 mA h g_sulfur_^−1^), which is superior to that of 2DPAV-TPB-BPZ electrode (794 mA h g_sulfur_^−1^) and the reported sulfur electrodes based on COFs or porous organic polymers tested under similar conditions (typically in the range of several hundreds of mA h g_sulfur_^−1^ for sulfur loading >70 wt%, see details in Table S3†). Both electrodes present high rate performance with a capacity retention of *ca.* 58% at 2C compared to that at 0.1C (Fig. 5c and S32†), suggesting that the host–guest interaction enables quick activation of the sulfur moieties at high current densities. The electrochemical impedance spectra are shown in Fig. S33.† The cycling stability was further evaluated at 0.5C for 200 charge–discharge cycles. The capacity decay rates of S_8_@2DPAV-TPT-BPZ and S_8_@2DPAV-TPB-BPZ are 0.064% and 0.129% per cycle, respectively (Fig. 5d), which contribute to excellent capacity retention of 80.1% and 73.0%, respectively.

## Conclusions

In conclusion, we have demonstrated two novel electron-deficient 2D PAVs as hosts for effective sulfur/polysulfide confinement. The developed 2D PAVs are chemically robust and remain highly crystalline after the sulfurization. Experimental results and theoretical calculations indicate that a BPZ-based electron-deficient polymer backbone strengthens the intermolecular interaction between 2D PAVs and the confined sulfur/polysulfides in the porous channels, which enables quick activation of the sulfur moieties and stabilizes polysulfides during electrochemistry. Consequently, the fabricated electrodes from sulfur encapsulated by 2D PAVs provide high capacity and excellent cycling stability. Our work highlights the great potential of developing electron-deficient 2D conjugated polymers for high-performance electrochemical applications, such as in metal–sulfur or chalcogen-based batteries. For the future development, various electron-deficient building blocks such as benzothiadiazoles, naphthalene diimides, diketopyrrolopyrroles, *etc.* should be explored to tune the electron deficiency of 2D PAVs towards n-type semiconductors. In addition, their pore sizes should be further engineered to boost the host–guest (sulfur, halogen, electrolyte, *etc.*) interaction for high-performance applications.

## Data availability

The data supporting this article have been included as part of the ESI.†

## Author contributions

X. F. and M. W. conceived and designed the project. A. L. W. performed most of the experiments and interpreted the data under the supervision of M. W. M. Z., S. H., J.-Q. H. and S. K. conducted the battery tests and analyzed the data. K. M. G. A. and A. S. helped with the solid-state UV-vis and Raman measurements. X. L. and P. K. contributed to DFT calculations. S. P. and E. B. measured the solid-state NMR spectra. F. A. helped with the Pawley refinements. A. M. contributed to TEM measurements. A. L. W., M. W. and X. F. co-wrote the paper with contributions from all co-authors.

## Conflicts of interest

There are no conflicts to declare.

## Supplementary Material

SC-OLF-D4SC06903J-s001
