# Supplementary material for: Electron-deficient two-dimensional poly(arylene vinylene) covalent organic frameworks: efficient synthesis and host–guest interaction
Source: Chem Sci. 2025 Feb 3;16(9):4152–8. doi: 10.1039/d4sc06903j (PMC11788921; doi:10.1039/d4sc06903j)
Supplement: SC-016-D4SC06903J-s001 [file SC-016-D4SC06903J-s001.pdf]

Supplementary Information (SI) for Chemical Science.
This journal is © The Royal Society of Chemistry 2025

Supplementary Information

Electron-Deficient Two-Dimensional Poly(arylene vinylene) Covalent Organic Frameworks: Efficient Synthesis and Host-Guest Interaction

Albrecht L. Waentig,# Xiaodong Li,# Meng Zhao,# Sattwick Haldar, Philomene Koko, Silvia Paasch, Alina Mueller, Karen M. Garcia Alvarez, Florian Auras, Eike Brunner, Andreas Schneemann, Jia-Qi Huang, Stefan Kaskel, Mingchao Wang,* and Xinliang Feng,**

Table of Contents

Section A: Methods and Equipment

Section B: Synthetic Procedures

Section C: Supplementary Figures

Section D: Supplementary Tables

Section E: Supplementary References

Section A: Methods and Equipment

General: Commercially available reagents and dry solvents were used without further purification if not otherwise stated. Reactions were performed using conventional vacuum and Schlenk technique. Flash column chromatography was performed using silica gel with a particle size range from 0.063-0.2 mm from VWR. Thin layer chromatography was done with silica-coated aluminum sheets with fluorescence indicator F_{254} from Merck. Commercially available aldehydes 1,3,5-tris(4-formylphenyl)benzene (TFPB) and 4,4',4''-(1,3,5-triazine-2,4,6-triyl)tribenzaldehyde (TTTB) were obtained from BLD-Pharmtech, Kaiserslautern, Germany.

Nuclear magnetic resonance (NMR) spectroscopy: The liquid-state ^{1}H- and ^{13}C-NMR spectra were recorded on a BRUKER AVANCE III 300 spectrometer at 25 °C using standard pulse technique. Chemical shifts are reported as δ-values in ppm. Solid-state ^{13}C CP-MAS NMR spectra of the obtained 2D PAVs were recorded with a BRUKER Ascend 800 MHz spectrometer at a resonance frequency of 201.2 MHz with a commercial double resonance 1.3 MAS NMR probe operating at MAS frequency of 50 kHz. Ramped ^{1}H-^{13}C cross-polarization (contact time: 4 ms, pulse repetition time: 3 s) and SPINAL ^{1}H-decoupling were applied.

Mass spectroscopy (MS): Mass spectra were recorded with a XEVO-G2XS -QTOF ESI-MS device with the samples dissolved in acetonitrile.

Powder X-ray diffraction (PXRD): PXRD patterns were recorded with a STOE STADI P diffractometer with Cu K_{α} radiation (α = 1.5406 Å) between 2θ = 2-30°. A custom-built stainless-steel holder was used, PXRD patterns were recorded in transmission mode.

UV-Vis absorption and fluorescence spectroscopy: An Agilent Cary 5000 UV-Vis-NIR, equipped with an integration sphere was used to obtain UV-Vis absorption spectra of the 2D PAVs dispersed in 2-propanol by ultrasonication for 5 minutes. Fluorescence emission spectra

were recorded with a PerkinElmer LS-55 spectrometer with the same dispersion. Solid state UV-Vis data was acquired on a same spectrometer equipped with a “Praying Mantis” solid state sample stage.

Gas physisorption measurements: Nitrogen physisorption measurements were performed at 77 K maintained by a liquid nitrogen bath with a Quantachrome Instruments Quadrasorb. With non-local density functional theory mode l (NLDFT, cylindrical pore model), pore sizes were calculated from the adsorption branch. Relative pressure ranges 0.05-0.2 were used for calculation of BET surface area. Prior to physisorption measurements, the samples were activated by supercritical carbon dioxide (scCO_2) using a Leica CPD 300 (40 °C, ca. 78 bar, 20 cycles) followed by vacuum activation at r.t. for 1 h.

Fourier-transform Infrared (FT-IR) spectroscopy: A Bruker Tensor II spectrometer with a universal ATR (attenuated total reflection) accessory was used to record background corrected FT-IR spectra in the range between 400−4000 cm^{-1}.

Raman spectroscopy: A Bruker RFS 100 FT-RAMAN spectrometer with 1064 nm laser wavelength and a nitrogen-cooled Ge-detector with spectral resolution of 1 cm^{-1} was employed.

Thermogravimetric analysis (TGA): TGA were performed on a TG50 analyzer (Mettler-Toledo) under argon flow 240 mL/min. with a heating rate of 5 ºC/min between 20-800 °C.

Scanning electron microscopy (SEM): SEM images were obtained with a Zeiss DSM 950 scanning electron microscope operated at 10 kV. The as prepared materials were dropcast from 2-propanol on silicon substrates and sputtered with gold.

Elemental analysis (EA): EA (CHNS) were obtained from a HEKATECH EA 3000 EURO VECTOR.

Pawley refinement: Pawley refinement was done with the Accelrys 2020 package. Starting parameters for Pawley refinement were: **2DPAV-TPT-BPZ**: a = b = 44.11 Å, c = 3.61 Å; $\alpha = \beta =$

90 °, γ = 120 °; for **2DPAV-TPB-BPZ**: a = 45.09 Å, b = 44.82 Å, c = 3.74 Å; α = 101.20 °, β = 74.93 °, γ = 121.36 °.

DFT calculation details: The first-principles calculations were carried out with the Vienna ab initio simulation package (VASP).[1] The interaction between ions and valence electrons is described using projector augmented wave (PAW) potentials, and the exchange-correlation between electrons is treated through using the generalized gradient approximation (GGA) in the Perdew-Burke-Ernzerhof (PBE) form.[2] DFT-D3 method was employed to calculate the van der Waals interaction.[3] To achieve the accurate simulations, the plane wave cutoff energy was 450 eV, a Gamma centered k-point grid of 1 × 1 × 11 and 1 × 1 × 1 was used for bulk and slab models. Ionic relaxations were carried out under the conventional energy (10^{-4} eV) and force (0.03 eV/Å) convergence criteria. HOMO and LUMO are obtained from the calculation of energy band structure. Adsorption energies $E_{adsorption}$ are given with reference to the isolated surface $E_{surface}$ relaxed upon removing the molecule from the unit cell using identical computational parameters and the energy of the molecule $E_{molecule}$.

$$E_{adsorption} = E_{molecule\ on\ surface} - E_{surface} - E_{molecule}$$

Preparation of 2D PAV-S_8 composites: 20 mg 2D PAV powders were mixed with 50 mg sulfur powders and hand-grinded for 30 min. Then, the mixture was transferred into a glass tube and sealed under vacuum. The sealed tube was transferred into the oven and heated under 155 °C for 6 h with a heating rate of 2 °C/min. The 2D PAV-S_8 composite containing 60 wt% S_8 was obtained after cooling to ambient temperature. For the composite containing 86 wt% S_8, 30 mg respective 2D PAV and 200 mg sulfur powders were used.

Preparation of sulfur cathode: 42 mg 2D PAV-S_8 composite, 5.6 mg super P, 5.6 mg multi-wall CNTs (MWCNTs), 2.8 mg polyvinylidene fluoride (PVDF, dissolved in N-methylpyrrolidone with a concentration of 0.02 mg/µL) were mixed and ground in NMP solvent for 30 min on a ball

mill to make a cathode slurry. The weight ratio of 2D PAV composite/carbon additives/binder is 75:20:5. Afterwards, the slurry was blade coated on Al foil and dried at 60 °C for 12 h. The sulfur loading was about 0.7 mg/cm^2. After cooling down, the coated Al foil was cut into small disks with a diameter of 12 mm to obtain sulfur electrodes for coin cells. The electrolyte/sulfur ratio is 49 µL/mg.

Assembly and cycling of Li-S battery: Li-S coin cells were assembled using standard 2025 coin cell, where lithium foil was the anode and the PP membranes served as separators. 1 mol/L LiTFSI and 5% LiNO3 dissolved in 1,3-dioxolane/ 1,2-dimethoxyethane (v/v=1:1) mixed solvents was adopted as the electrolyte. 5 and 20 µL electrolyte was added to the anode and cathode side in each cell. The cells were galvano-statically cycled between 1.7–2.8 V on a LAND battery cycler under different C-rates (1C=1672 mA/g). CV tests were conducted on a Biologic electrochemical workstation at a scanning rate of 0.1 mV/s, and the EIS test was also conducted on the same workstation with potentiostatic mode at open circuit potential over a frequency range from 0.1 Hz to 10k Hz.

Preparation of Li_2S_6 solution: A Li_2S_6 solution was prepared by mixing 46 mg Li_2S (1 mmol) with 160 mg sulfur (5 mmol) in a Schlenk tube equipped with a stirring bar. Next, 10 mL of a 1:1 mixture of diethylene glycol dimethyl ether (DME) and 1,3-dioxolane (DOL) were added and the suspension was stirred under inert conditions over night. A thick red solution was formed during this process, which was stored in the glovebox until further use.

Preparation of 2D PAV-S_6^{2-} composites for RAMAN and UV-Vis spectroscopies: The Li_2S_6 solution was diluted to 0.1 M with DME/DOL (1:1) as the solvent in a glovebox. **For RAMAN measurement,** glass capillaries containing the 2D PAVs were soaked with 0.1 M Li_2S_6 solution in the glovebox at 25°C for 5 minutes. The capillaries were sealed with hot wax under inert atmosphere. The samples were then transferred out of the glove box for immediate RAMAN

spectroscopy study. **For solid-state UV-visible absorption measurements,** the 2D PAVs were soaked in 0.1 M Li_2S_6 solution in the glovebox at 25°C for 5 minutes. The soaked 2DPAVs were transferred out of the glovebox to the solid-state UV-vis spectrometer with a sealed UV-Vis measurement cell and measured immediately.

Section B: Synthetic Procedures

Synthesis of 5,5'-dimethyl-2,2'-bipyrazine (DMBP)

A 100 mL Schlenk-flask was charged with 20 mL DMF and $NiCl_2 \cdot 6H_2O$ (0.49 g, 1.73 mmol, 0.1 eq.) under nitrogen atmosphere. The solution was heated to 50 °C following the addition of 2-bromo-5-methylpyrazine (3 g, 17.34 mmol, 1 eq.), LiCl (0.74 g, 17.34 mmol, 1 eq.) and zinc powder (1.36 g, 20.81 mmol, 1.20 eq.). The mixture stirred for 5 more minutes, then a grain of iodine and 0.2 mL of concentrated AcOH were added to the mixture. The mixture was stirred at 50 °C for 5 h and monitored by thin layer chromatography. After complete conversion, DMF was removed under reduced pressure. 2 mL concentrated aqueous HCl were added to dissolve remaining zinc. The mixture was brought to pH 8-9 with aqueous ammonia, following the addition of 4 g ethylenediaminetetraacetic acid. It was extracted 4 times with 30 mL dichloromethane (DCM) and the organic phase was collected, dried over $MgSO_4$, and concentrated in vacuo. Purification of the crude was performed by flash chromatography eluting with DCM/ethyl acetate/triethylamine (1:1:0.01) to give 0.8 g (50%) **DMBP** as a white powder.

NMR: ^{1}H NMR (300 MHz, CD_2Cl_2) δ = 9.29(s, 2 H), 8.40(s, 2 H), 2.52(s, 6 H). ^{13}C NMR (76 MHz, CD_2Cl_2) δ = 154.17, 146.79, 143.20, 141.71, 20.81.

ESI-MS: measured m/z [M+H] = 187.0994; calculated m/z [M+H] = 187.0994

Synthesis of model compound 5,5'-di((E)-styryl)-2,2'-bipyrazine (DSBPZ)

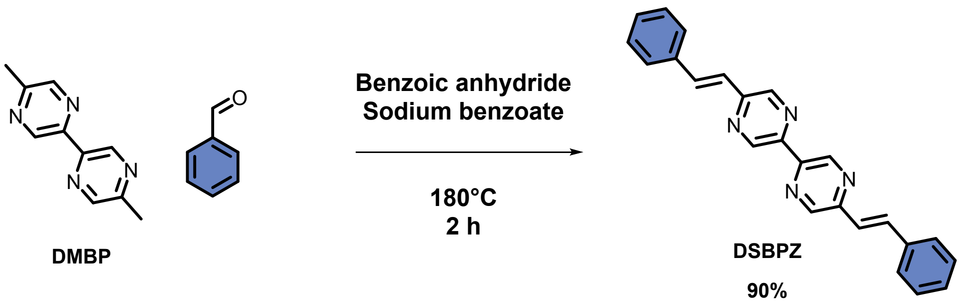

Scheme S1: Synthesis scheme of model compound **DSBPZ.**

In a borosilicate glass ampule, 20 mg **DMBP** (0.1 mmol, 1 eq.), benzyl aldehyde (55 µL, 0.5 mmol, 5 eq.) 70 mg benzoic anhydride and 30 mg sodium benzoate were combined. The mixture was degassed by 2 freeze-pump and thaw cycles and the ampule was flame-sealed. The reaction was heated for 24 h in a sand bath at 180 °C. After cooling to r.t. the product was washed subsequently with water and methanol, followed by drying under reduced pressure to afford 35 mg (90%) **DSBPZ** as a yellow powder.

NMR: ^{1}H NMR (300 MHz, $C_2D_2Cl_4$) δ= 9.47(s, 2 H), 8.67(s, 2 H),7.78 (d, J = 15.9 Hz, 2H), 7.60 (d, J = 7.9 Hz, 4H), 7.44 – 7.28 (m, 6H), 7.20 (d, J = 16.0 Hz, 2H). Due to the low solubility of **DSBPZ** in common organic solvents, only ^{1}H NMR could be obtained.

ESI-MS: measured m/z [M+H] = 363.1612; calculated m/z [M+H] = 363.1610

Kinetic studies

Based on our findings for good crystallinity when employing sodium benzoate **(SBz**) together with benzoic anhydride (**BAn**) for our systems which we found to give good crystallinity in contrast to only using benzoic acid (**BAc**) or **BAn**/**BAc** mixtures, influence of **SBz/BAn**-mixture on the formation of model compound 5,5'-di((E)-styryl)-2,2'-bipyrazine was studied.

In pyrex tubes under nitrogen atmosphere, 20 mg **DMBP** (0.1 mmol, 1 eq.) and benzyl alcohol (22 µL, 0.2 mmol, 2 eq.) were combined. For **BAn**: 60 mg benzoic anhydride, for **BAn/BAc**: 60 mg benzoic anhydride and 30 mg benzoic acid and for **BAn/SBz**: 60 mg benzoic anhydride and 30 mg sodium benzoate were added. The different entries were heated under nitrogen atmosphere to 180 °C, after 5 minutes, 30 minutes, 60 minutes and 120 minutes, samples were taken under nitrogen atmosphere from each entry and dissolved in 0.6 mL deuterated dichloromethane for ^{1}H-NMR spectroscopy.

For each entry and ^{1}H spectrum, integrals of the proton signal of **DMBP** methyl group (~2.6 ppm) were compared with the integration of aromatic signals between 7 and 9 ppm to find the relative decrease of DMBP methyl group as a measure for reaction progress (Figure S5).

Synthesis of 2DPAV-TPB-BPZ

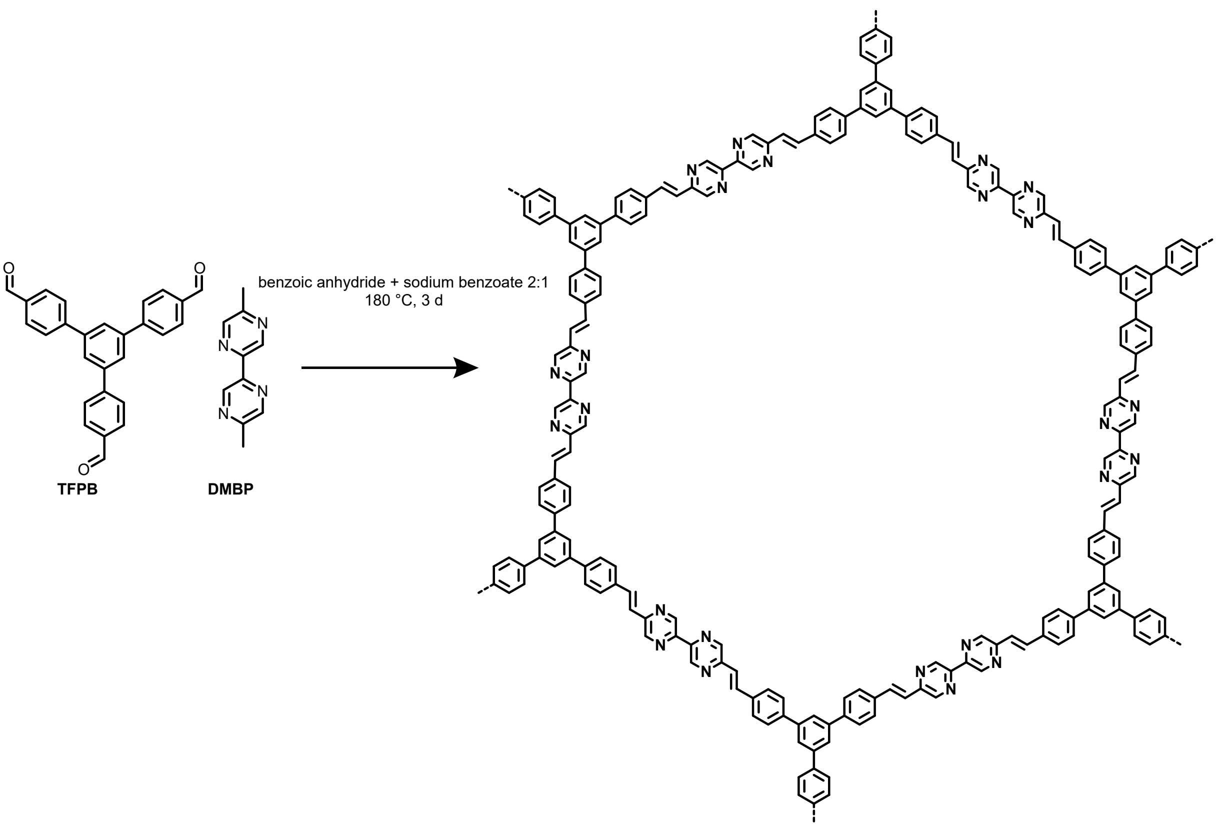

Scheme S2: Synthetic route towards **2DPAV-TPB-BPZ.**

5'-(4-formylphenyl)-[1,1':3',1''-terphenyl]-4,4''-dicarbaldehyde (**TFPB**) (10 mg, 25.6 µmol, 1 eq.) and **DMBP** (7.15 mg, 38 µmol, 1.5 eq.) were added in a glass ampule. 30 mg benzoic anhydride and 15 mg sodium benzoate were added to the ampule. The ampule was evacuated at r.t. for 30 min and was flame-sealed and heated in a sand bath at 180 °C for 3 d. After cooling to r.t. the formed yellow solid was ground in an agate-mortar and repeatedly washed with acetone, water, DMF and DCM. The resulting solid was dried under vacuum to afford **2DPAV-TPB-BPZ** as orange powder (13.3 mg, 85 %).

Synthesis of 2DPAV-TPT-BPZ

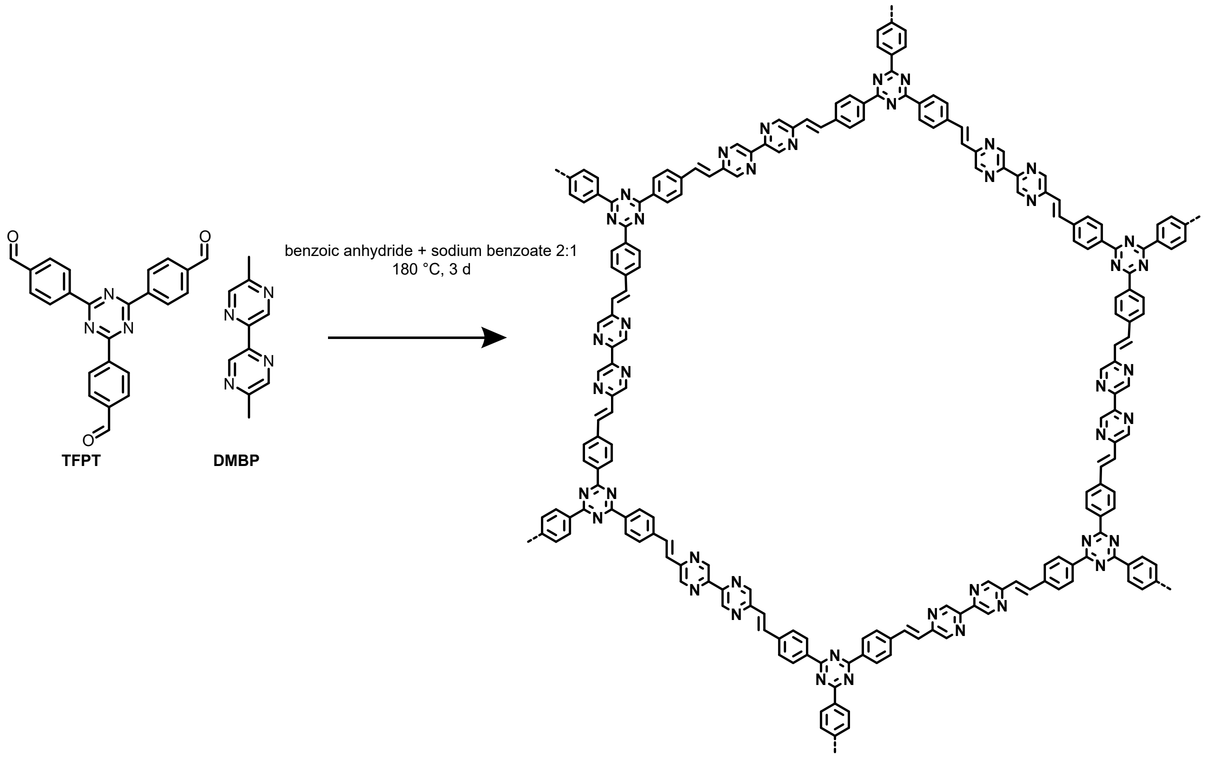

Scheme S3: Synthetic route towards **2DPAV-TPT-BPZ**.

4,4',4''-(1,3,5-triazine-2,4,6-triyl)tribenzaldehyde (**TTTB**) (10 mg, 25,4 µmol, 1 eq.) and **DMBP** (7.1 mg, 38.1 µmol, 1.5 eq.) were added in a glass ampule. 30 mg benzoic anhydride and 15 mg sodium benzoate were added to the ampule. The ampule was evacuated at r.t. for 30 min and was flame-sealed and heated in a sand bath at 180 °C for 3 d. After cooling to r.t. the formed yellow solid was ground in an agate-mortar and repeatedly washed with acetone, water, DMF and DCM. The resulting solid was dried under vacuum to afford **2DPAV-TPT-BPZ** as yellow powder (13.8 mg, 89 %).

Synthesis of 2DPAV-TPB-BP

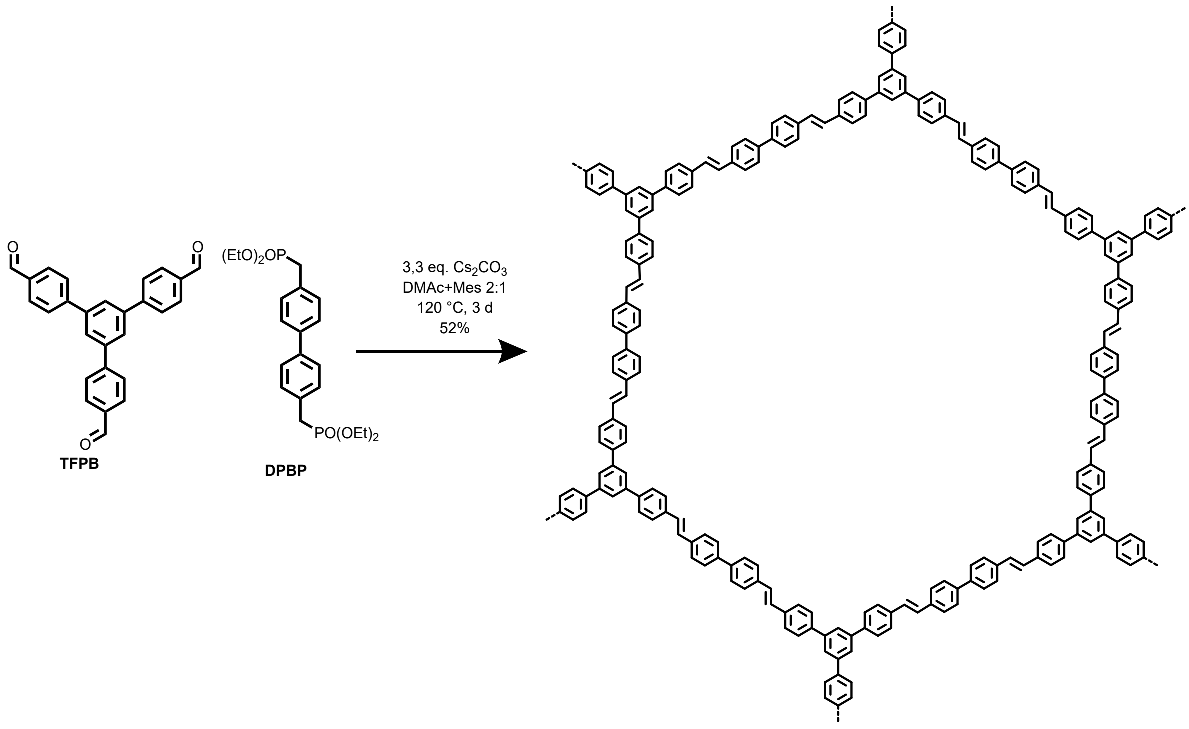

Scheme S1: Synthesis of **2DPAV-TPB-BP**.

To 5'-(4-formylphenyl)-[1,1':3',1''-terphenyl]-4,4''-dicarbaldehyde (**TFPB**, 20 mg, 51.2 µmol, 1.0 eq.), 4,4'bis(diethyl-phosphonomethyl)biphenyl (**DPBP**) (35 mg, 76.8 µmol, 1.5 eq.) and Cs_2CO_3 (55.0 mg, 84.5 µmol, 3.3 eq.) were added in a glass ampule. 2 mL DMAc+ Mes 2:1 were added and the ampule was sealed under vacuum three freeze-pump and thaw cycles. Afterward, the ampule was heated for three days at 120 °C and subsequently cooled to room temperature. The precipitate was filtered and washed with acetone, water, aqueous HCl, water and ethanol. The obtained powder was filtered and dried under vacuum to afford **2DPAV-TPB-BP**.

Section C: Supplementary Figures

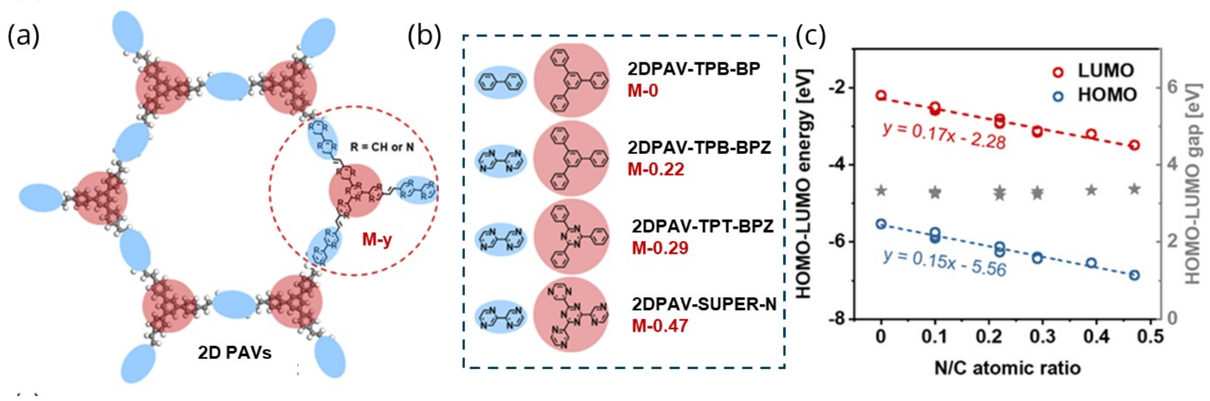

Figure S1: Tuning of electron-deficiency in honeycomb 2D PAVs by increasing the N/C ratio. a,b) Structural representation of 2D PAVs with the red dashed circle depicting the model compounds (see also Figure S2/S3) with defined N/C ratios. c) HOMO and LUMO energy levels in relation to their N/C atomic ratios. The dashed lines are linear fits of the dots. Figures S2 and S3 give further insights about different structures of model compounds.

To tune the energy levels and control the electron density in 2D PAVs, we selected 2D PAVs with a honeycomb lattice and designed 11 arylene-vinylene-based model compounds (M-y in Figure 1a, y is the N/C atomic ratio, see details below) with gradually increased N/C atomic ratio. Pyrazine and triazine units are employed to substitute the phenyl rings in the all-carbon-based M-0 to tune the electron affinity (y = 0, 0.10, 0.22, 0.29, 0.39 or 0.47; the chemical structures of M-0 and the representative M-0.22, M-0.29 as well as M-0.47 are shown in Figure S2 and Figure S3; one M-0, three different M-0.10, three different M-0.22, two different M-0.29, one M-0.39, and one M-0.47 molecules are considered for calculation). We calculated the energy levels of the HOMO and LUMO of M-y by the density functional theory (DFT) method. Although the 11 model compounds exhibit similar HOMO-LUMO gaps in the range of 3.21-3.37 eV, their HOMO/LUMO energy levels differ significantly from each other.

Despite of different N positions, various M-0.10 (or M-0.22/M-0.29) molecules possess almost identical HOMO/LUMO energies. Increasing the N/C atomic ratio from 0 to 0.29, the LUMO energy level is gradually lowered by ca. 0.35, 0.33, and 0.26 eV in M-0.10, M-0.22, and M-0.29, respectively (compared to M-0; HOMO energy is lowered by 0.30, 0.34, and 0.24 eV, respectively), which suggests a considerable electron deficiency in the pyrazine/triazine-based model compounds. Interestingly, such a decrease follows a linear relationship between the orbital energies and N/C atomic ratios; the HOMO/LUMO energies are further lowered by ca. 0.1 and 0.3 eV in M-0.39 and M-0.47, respectively. These results imply the great feasibility of modulating the energy levels by precise molecular design in the vinylene-linked model compounds and COFs.

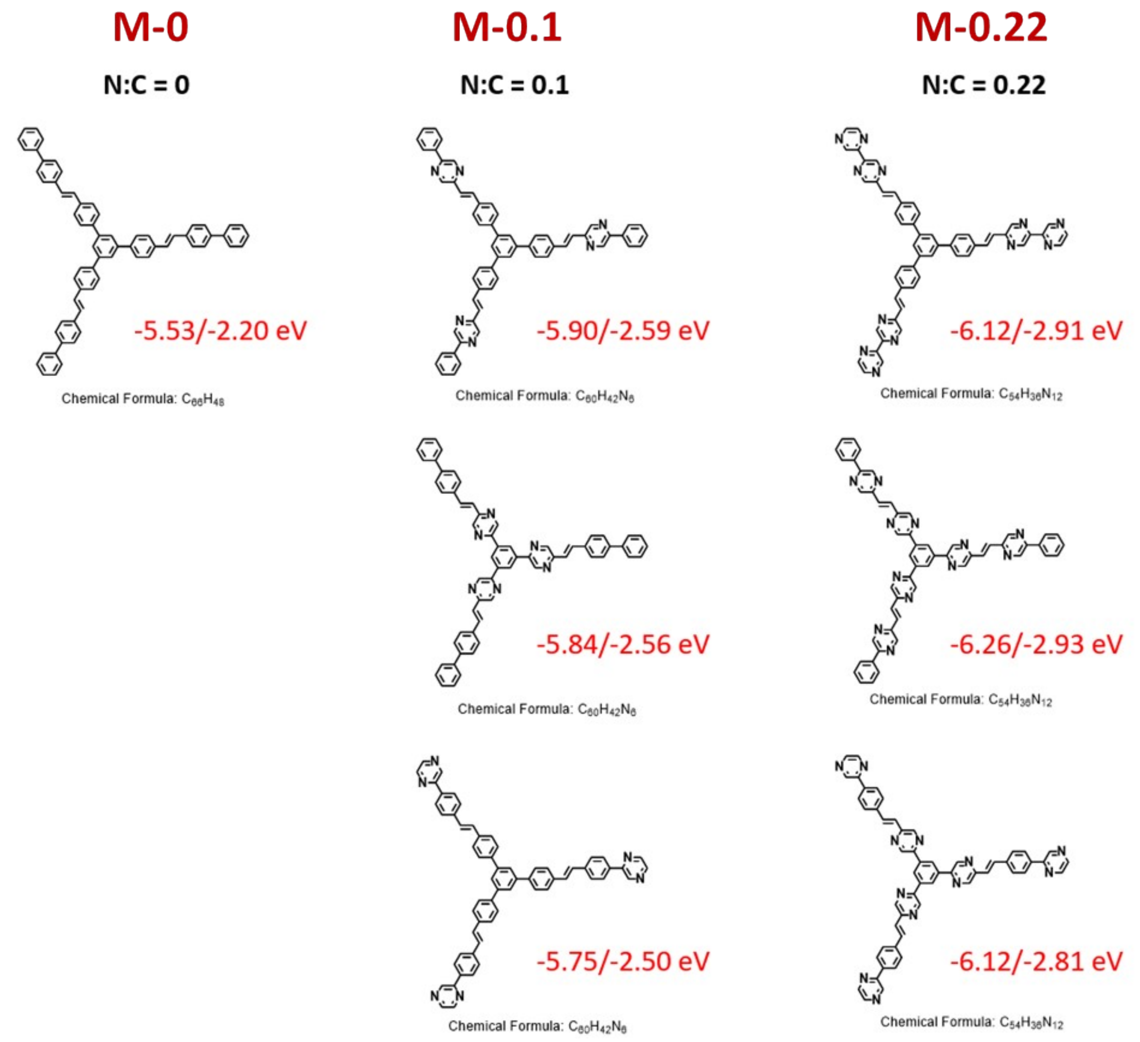

Figure S2: Chemical structures and HOMO/LUMO energy levels of the model compounds M-0, M-0.1 and M-0.22 with N:C atomic ratios in the range of 0–0.22. In model compounds with relatively low N:C atomic ratios, the pyrazine and triazine units are mostly not placed directly next to each other.

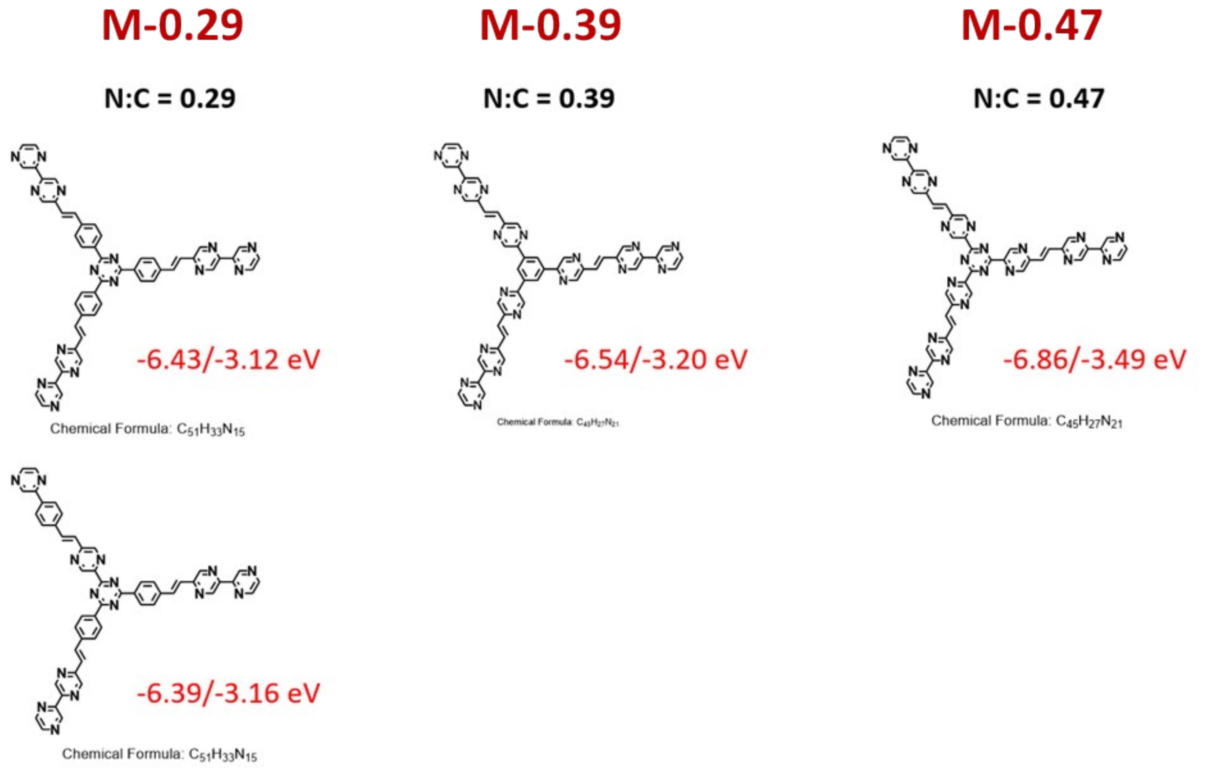

Figure S3: Chemical structures and HOMO/LUMO energy levels of the model compounds M-0.29, M-0.39 and M-0.47 with N:C atomic ratios in the range of 0.29–0.47.

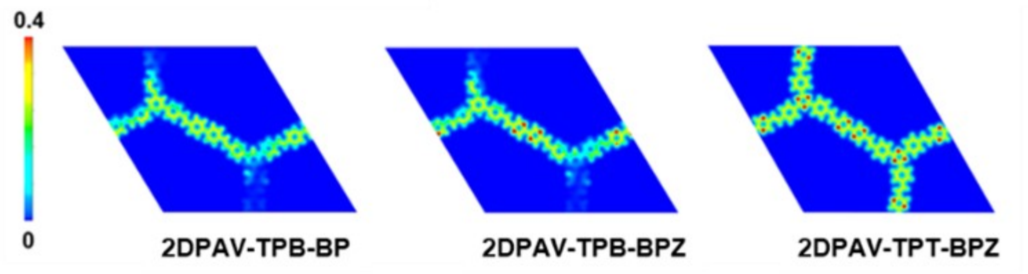

Figure S4: Electron density polarization in the synthesized 2D PAVs. Calculated overall electron density distribution.

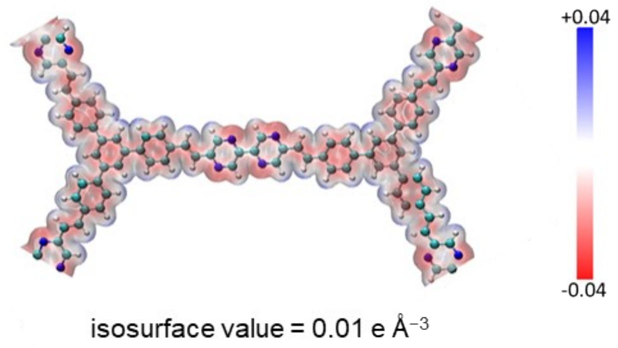

Figure S5: Electrostatic potential surfaces of **2DPAV-TPB-BPZ**.

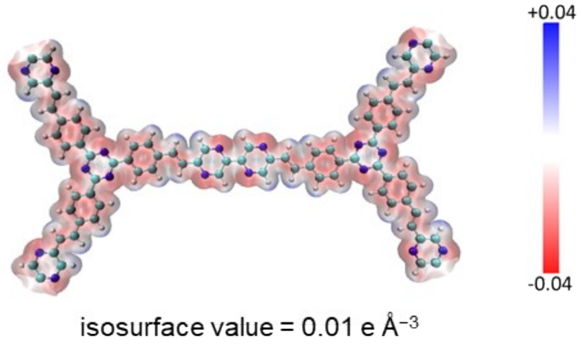

Figure S6: Electrostatic potential surfaces of **2DPAV-TPT-BPZ**.

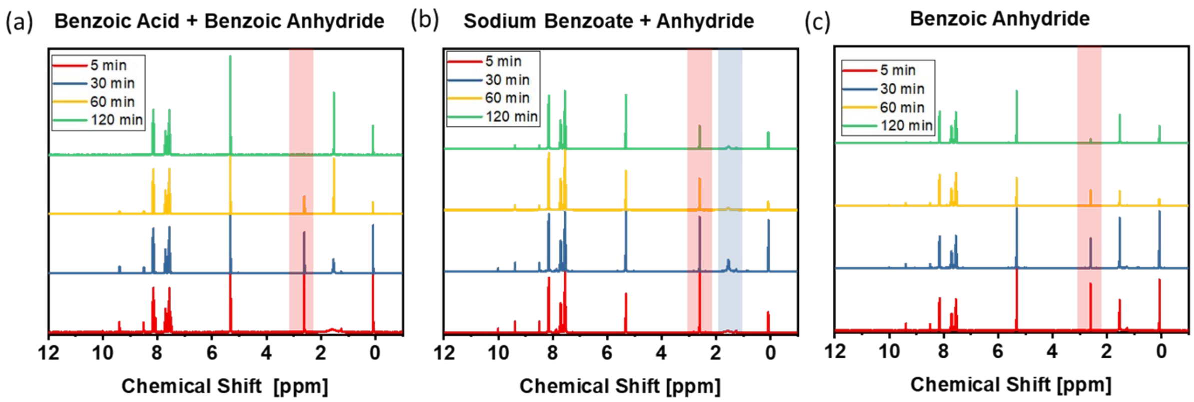

Figure S7: Time-dependent ^{1}H-NMR spectra for a) **BAn/BAc**, b) **BAn/SBz** and c) **BAn** highlighting the different consumption rates of the **DMBP** methyl group.

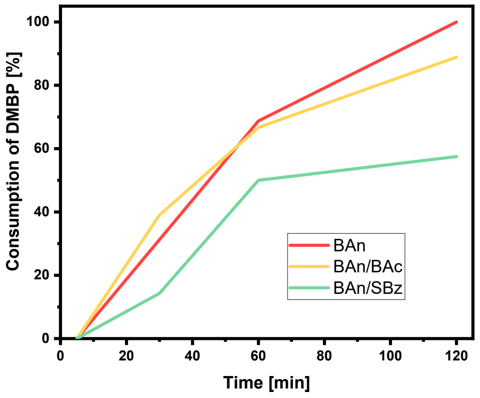

Figure S8: DMBP consumption deducted from the time dependent ^{1}H-NMR measurements. A strikingly lower reaction rate for **BAn/SBz** mixture is observed.

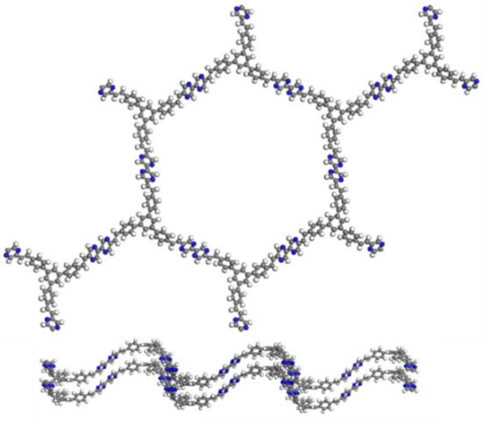

2DPAV-TPB-BPZ: Rwp= 4.40% Rp= 11.19%

Figure S9: Pawley-refined structure of **2DPAV-TPB-BPZ** in AA-stacking. View along the x-y-direction as well as along the z-direction. Space group P-3. Refined lattice constants a=b= 39.0 Å. Carbon atoms are displayed in grey, nitrogen atoms in blue and hydrogen atoms in white.

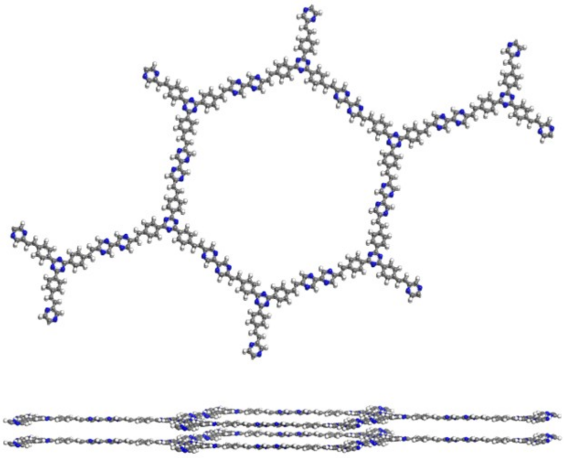

2DPAV-TPT-BPZ : Rwp= 4.36% Rp= 13.07%

Figure S10: Pawley-refined structure of **2DPAV-TPT-BPZ** in AA-stacking. View along the x-y-direction as well as along the z-direction. Space group P-3. Refined lattice constants a=b= 40.0 Å. Carbon atoms are displayed in grey, nitrogen atoms in blue and hydrogen atoms in white.

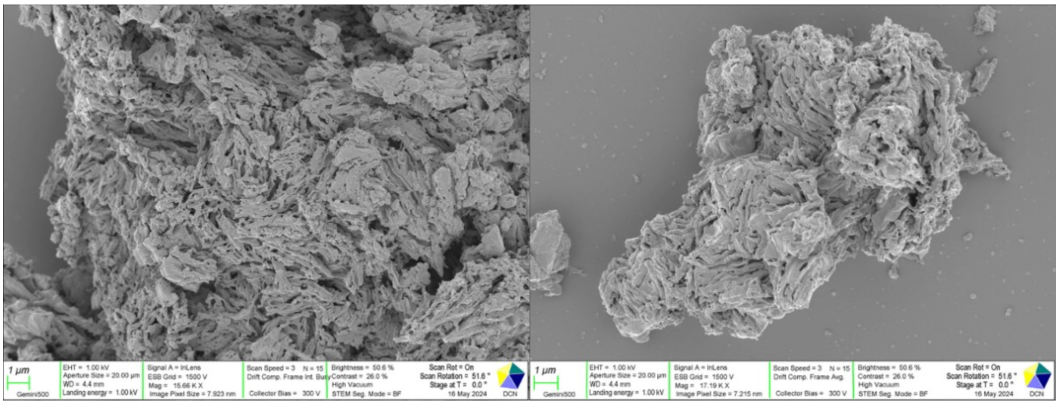

Figure S11: SEM micrographs of **2DPAV-TPB-BPZ**.

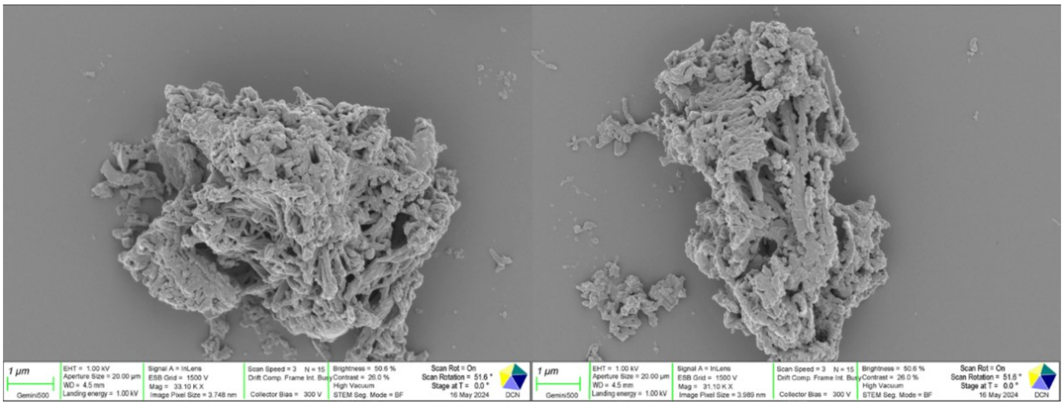

Figure S12: SEM micrographs of **2DPAV-TPT-BPZ**.

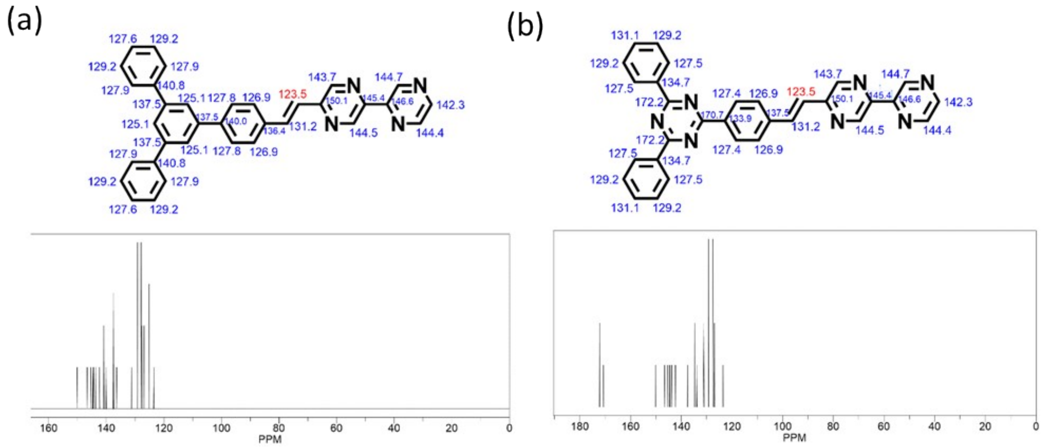

Figure S13: Predicted ^{13}C NMR of a) **2DPAV-TPB-BPZ** and b) **2DPAV-TPT-BPZ** using respective structural models by ChemDraw software.

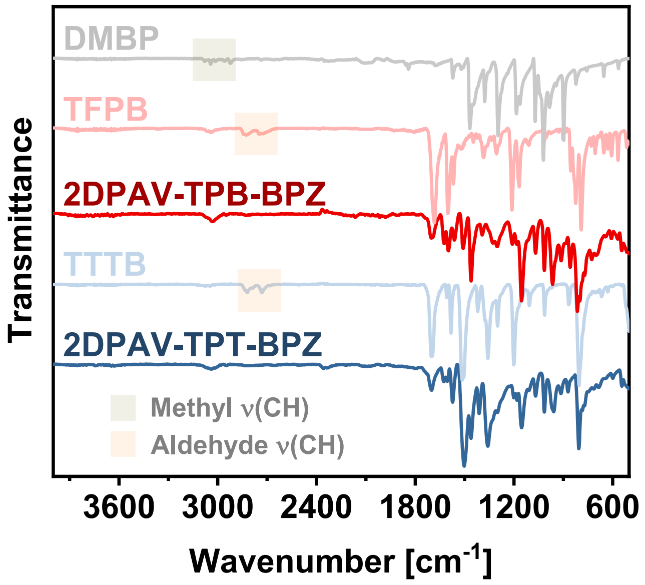

Figure S14: FTIR spectra of **2DPAV-TPB-BPZ** and **2DPAV-TPT-BPZ** in comparison with methyl DMBP monomer showing the successful consumption of aldehyde and methyl groups.

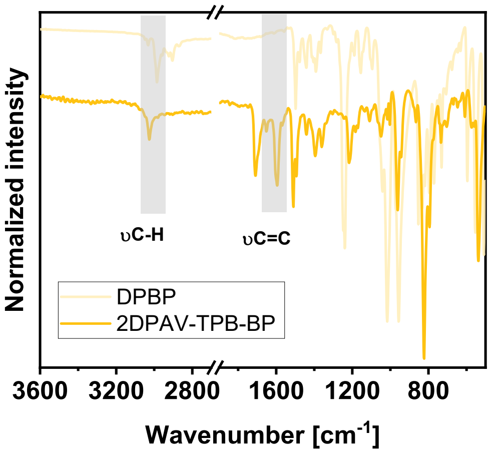

Figure S15: FTIR spectrum of **2DPAV-TPB-BP** and phosphonate monomer **DPBP**.

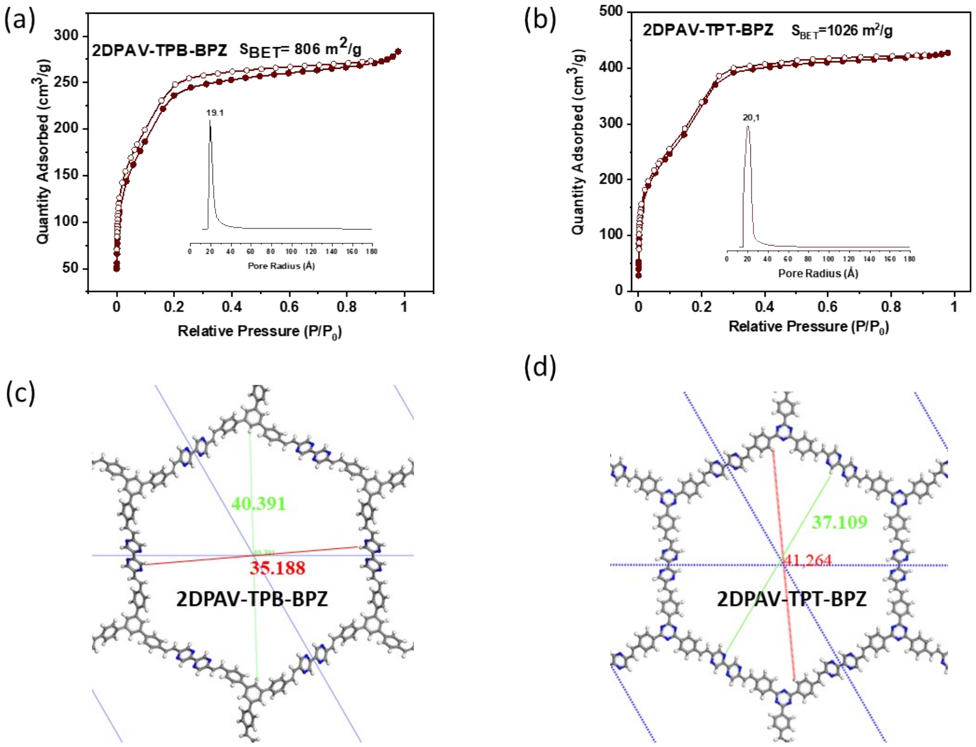

Figure S16: Nitrogen physisorption measurements at 77 K including fitted pore-radii for a) 2DPAV-TPB-BPZ, b) 2DPAV-TPT-BPZ. Filled and empty symbols represent ad- and desorption. The pore radii of 2D PAVs are 1.91 nm for **2DPAV-TPB-BPZ** and 2.01 nm for **2DPAV-TPT-BPZ**. The pore diameters are 3.82 nm for **2DPAV-TPB-BPZ** and 4.02 nm for **2DPAV-TPT-BPZ**. c,d) Pore information of the refined structures.

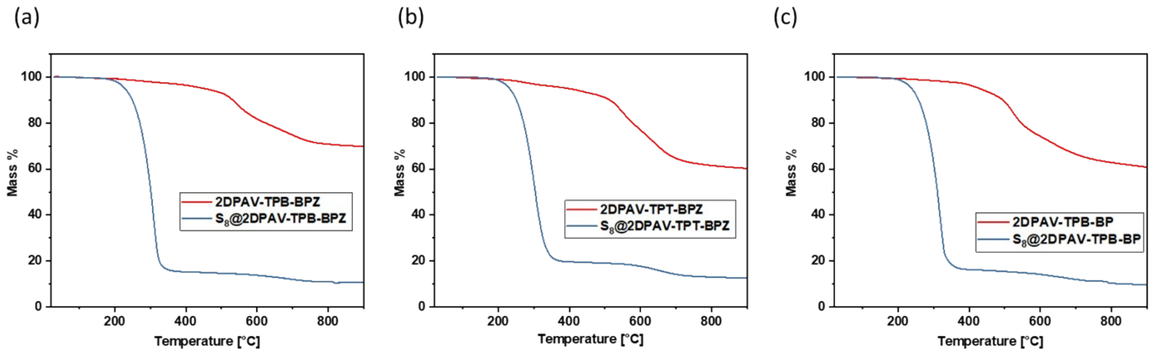

Figure S17: TGA analysis. a) Pristine and sulfurized **2DPAV-TPB-BPZ**. b) Pristine and sulfurized **2DPAV-TPT-BPZ**. c) Pristine and sulfurized **2DPAV-TPB-BP**. The data indicate a similar sulfur content of > 80 wt% in all composites.

.

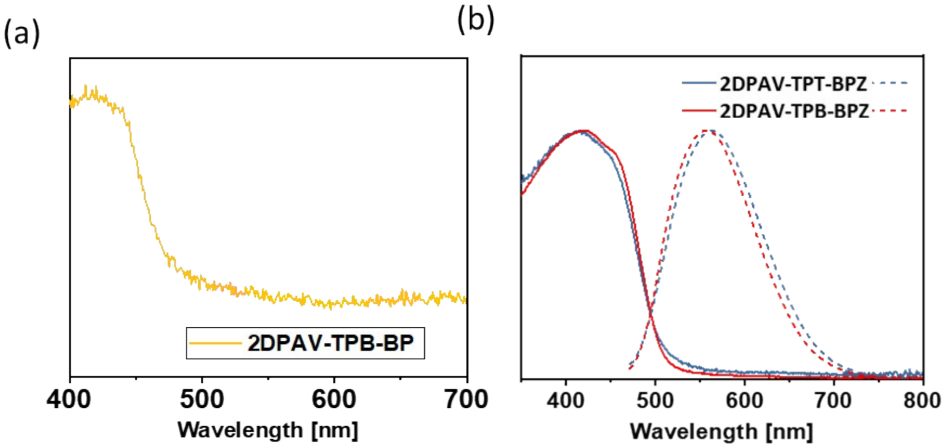

Figure S18: a) Solid-state UV-Vis absorption spectrum of **2DPAV-TPB-BP**. b) UV-Vis absorption/ fluorescence spectra of **2DPAV-TPB-BPZ** and **2DPAV-TPT-BPZ** in 2-propanol.

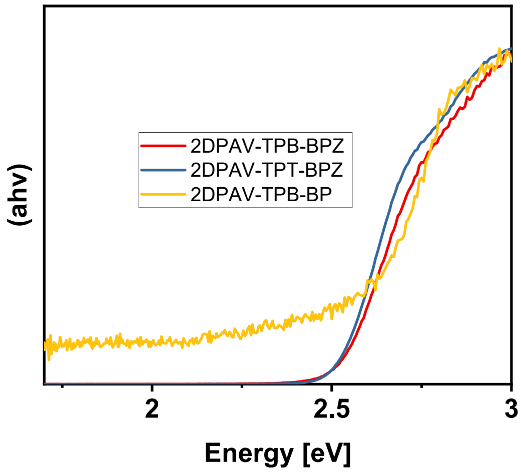

Figure S19: Tauc plots of **2DPAV-TPB-BPZ, 2DPAV-TPT-BPZ** and **2DPAV-TPB-BP**.

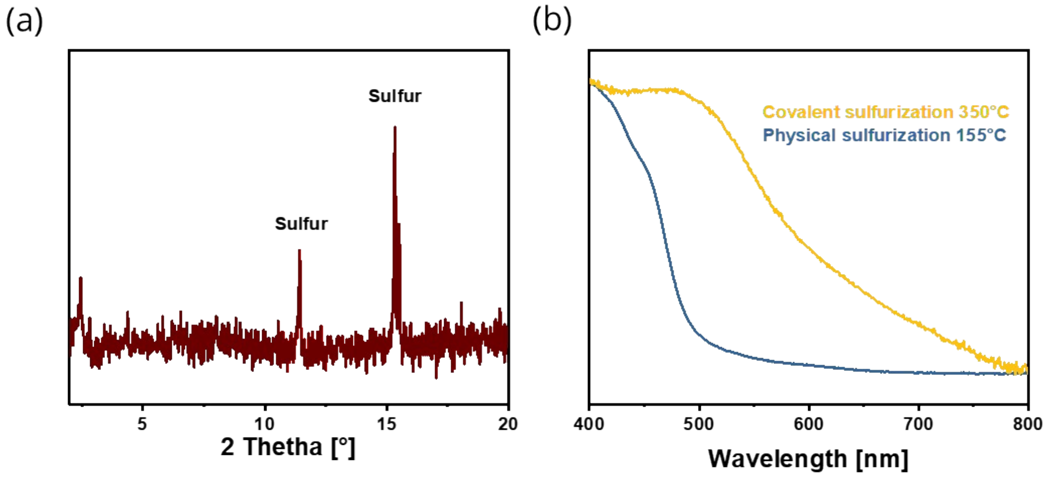

Figure S20: a) PXRD diffractogram of **S_8@2DPAV-TPT-BPZ** sulfurized at 350 °C for 12 h. The amorphization indicates a covalent bonding of sulfur to the material. b) UV-Vis absorption spectrum of covalently (350°C) and non-covalently (155°C) sulfurized **S_8@2DPAV-TPT-BPZ**. Significantly different absorption discloses covalent sulfurization at elevated temperatures.

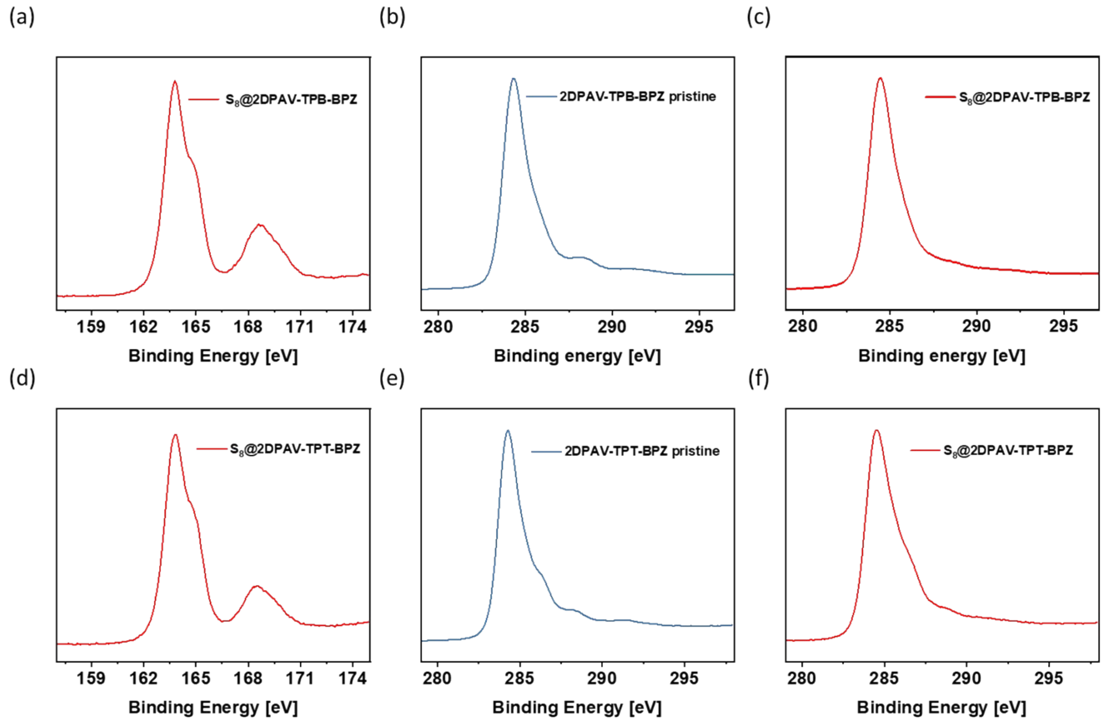

Figure S21: a), d) S 2p XPS spectra of **S_8@2DPAV-TPB-BPZ** and **S_8@2DPAV-TPT-BPZ.** b), e) C 1s XPS spectra of pristine **2DPAV-TPB-BPZ** and **2DPAV-TPT-BPZ**. c), f) C 1s XPS spectra of **S_8@2DPAV-TPB-BPZ** and **S_8@2DPAV-TPT-BPZ.**

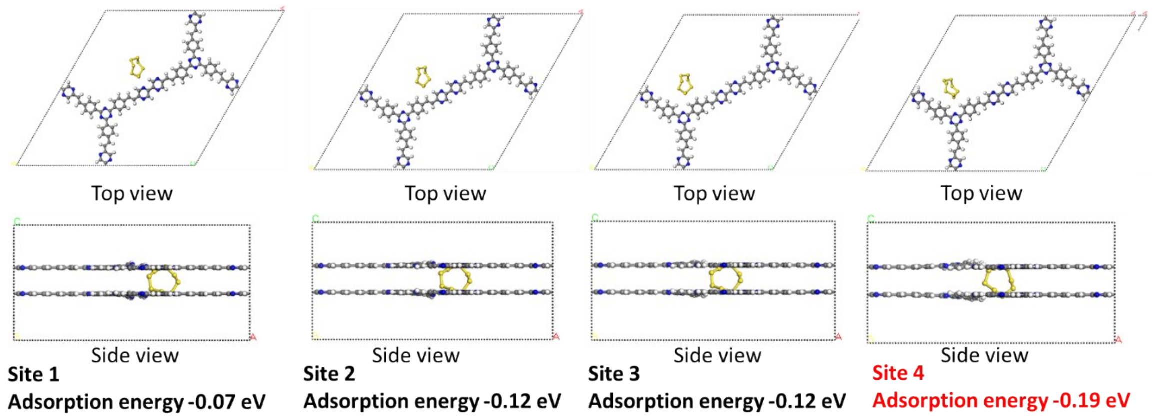

Figure S22: DFT Computational S_8 sulfur adsorption with the S_8-ring aligned vertically to the **2DPAV-TPT-BPZ** sheets on four different sites of an ideally calculated unit cell.

DFT calculations were performed to explore the most plausible S_8-adsorption sites. Taking **S_8@2DPAV-TPT-BPZ** as an example, the adsorption of one S_8 molecule near triazine (the most electron-deficient unit in **2DPAV-TPT-BPZ**, is energetically more favorable than that close to pyrazine or other moieties (–0.19 vs. ca. –0.07 eV). Considering the spatial size of the large S_8 molecule and to simplify the computation, we have placed the sulfur molecule vertically along the pore wall of bilayer 2D PAVs. Among the calculated four positions in the 2D PAVs, site 4 seems to be the most plausible site for sulfur adsorption.

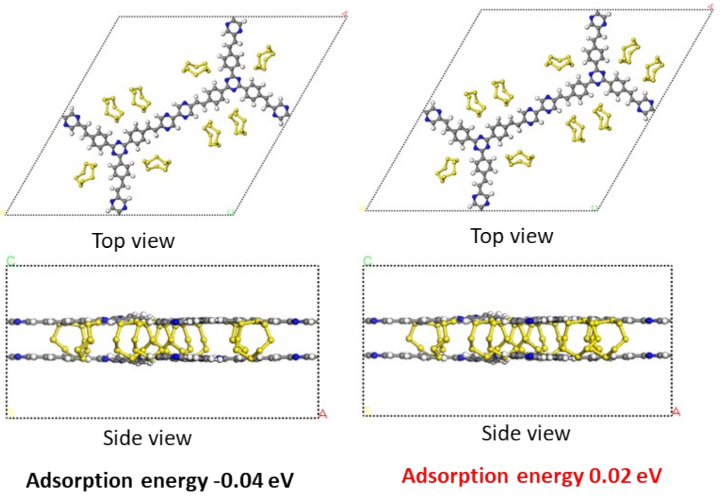

Figure S23: DFT Computational multi S_8 adsorption with 8 or 9 S_8-rings aligned vertically to the **2DPAV-TPT-BPZ**.

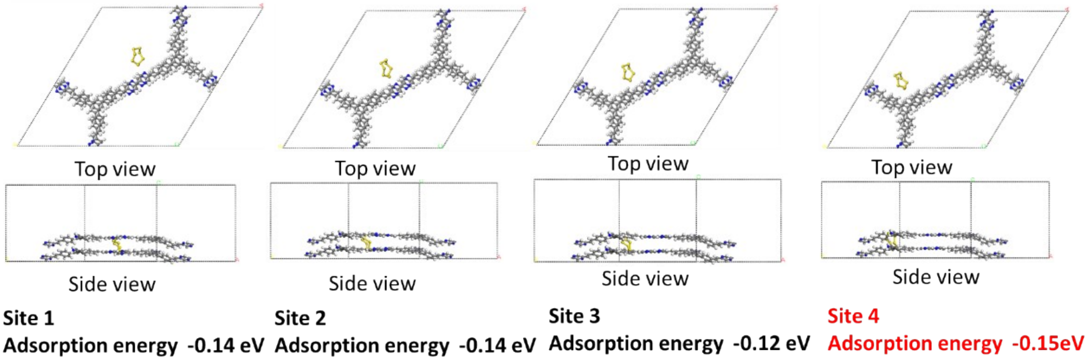

Figure S24: DFT Computational S_8 sulfur adsorption with the S_8-ring aligned vertically to the **2DPAV-TPB-BPZ** sheets on four different sites of an ideally calculated unit cell. **2DPAV-TPB-BPZ** does not show strongly preferred adsorption sites due to the absence of triazine units and it possesses higher adsorption energies (in the range of –0.12 ~ –0.15 eV,) than **2DPAV-TPT-BPZ**.

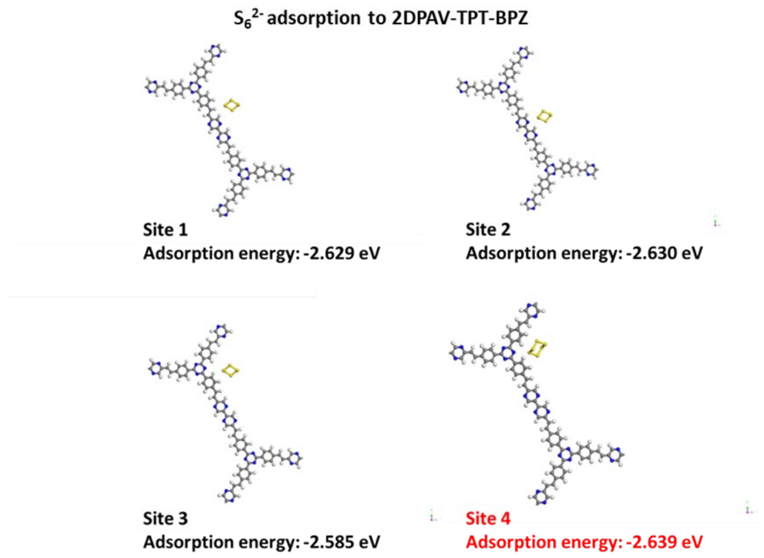

Figure S25: DFT Computational S_6^{2-} adsorption with the S_6^{2-} anion aligned vertically to the **2DPAV-TPT-BPZ** sheets on three different sites of an ideally calculated unit cell.

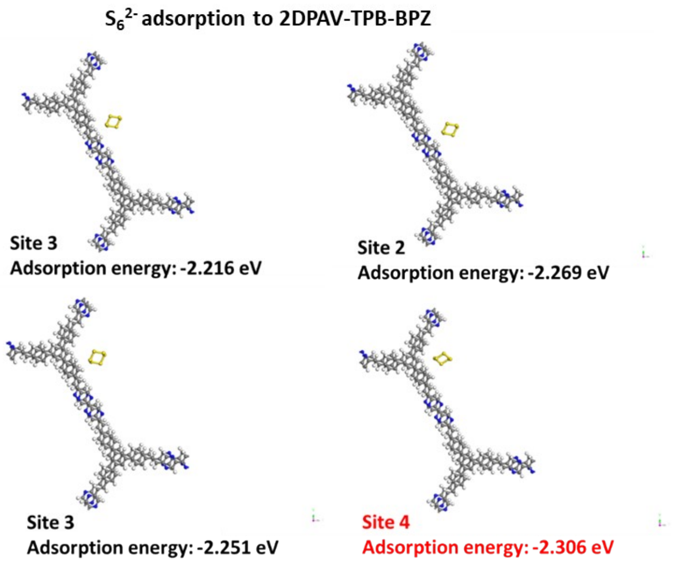

Figure S26: DFT Computational S_6^{2-} adsorption with the S_6^{2-}aligned vertically to the **2DPAV-TPB-BPZ** sheets on three different sites of an ideally calculated unit cell.

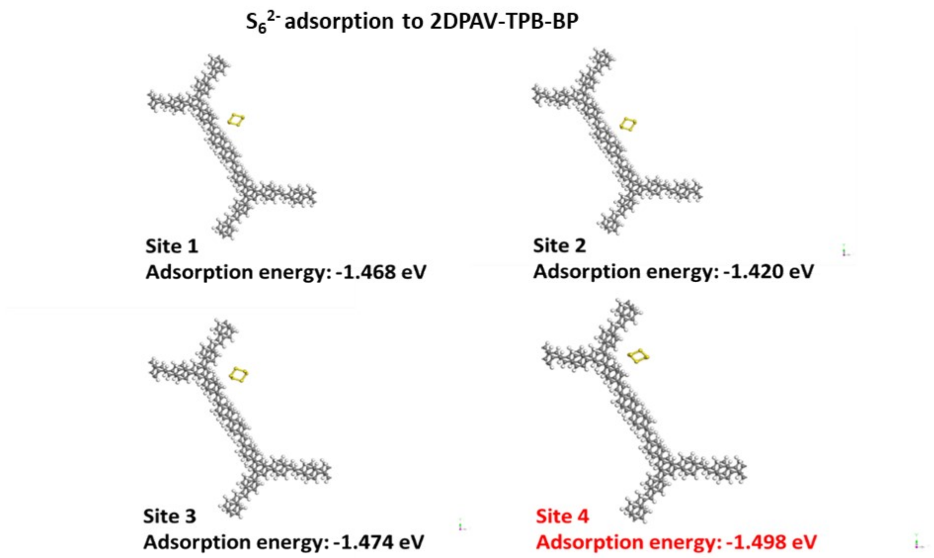

Figure S27: DFT Computational S_6^{2-} adsorption with the S_6^{2-}aligned vertically to the **2DPAV-TPB-BP** sheets on three different sites of an ideally calculated unit cell. The S_6^{2-} adsorption energy is the lowest for the three compared PAVs.

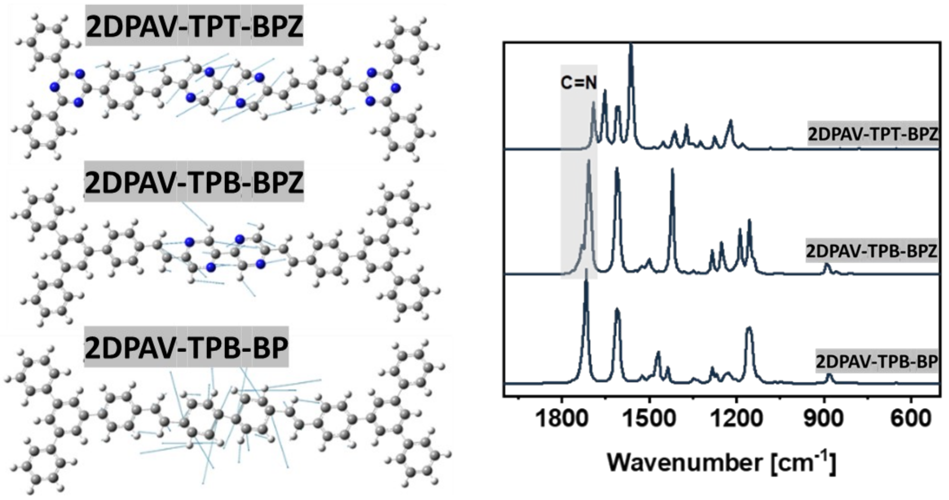

Figure S28: Calculated Raman vibrational modes and spectra of **2DPAV-TPT-BPZ**, **2DPAV-TPB-BPZ** and **2DPAV-TPB-BP**. For **BPZ-**based 2D PAVs, the peak on the left side corresponds the C=N vibrations; while for **2DPAV- TPB-BP**, the left peak stems from C=C vibrational mode.

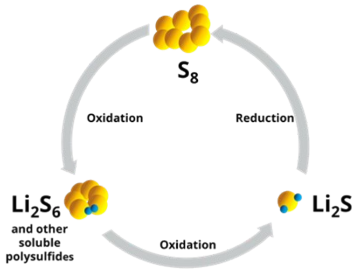

Figure S29: Schematic displaying the redox reactions of sulfur in Li-S batteries. Li_2S_6 is selected as the prototype soluble polysulfide.

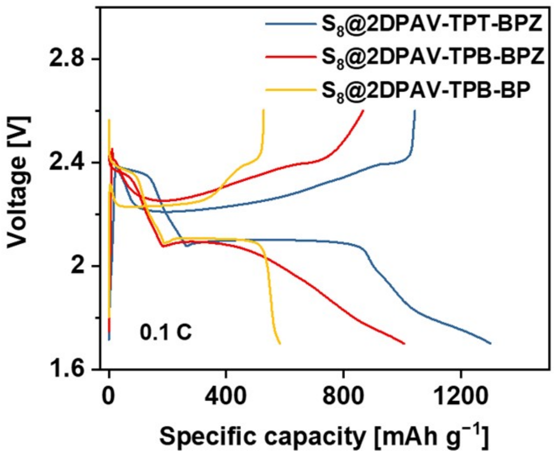

Figure S30: Charge-discharge profiles of the composite electrodes (60% Sulfur) in Li-sulfur batteries.

Taking S_8@2DPAV-TPT-BPZ as an example, the composite containing 60 wt% sulfur shows a discharge capacity of ~900 mAh/g_{sulfur} at 0.1 C, which is superior to that of ~800 mAh/g_{sulfur} for the sample with 86 wt% sulfur. Thus, more adsorbed sulfur results in weaker host-guest interaction thus lower Li-S battery performance.

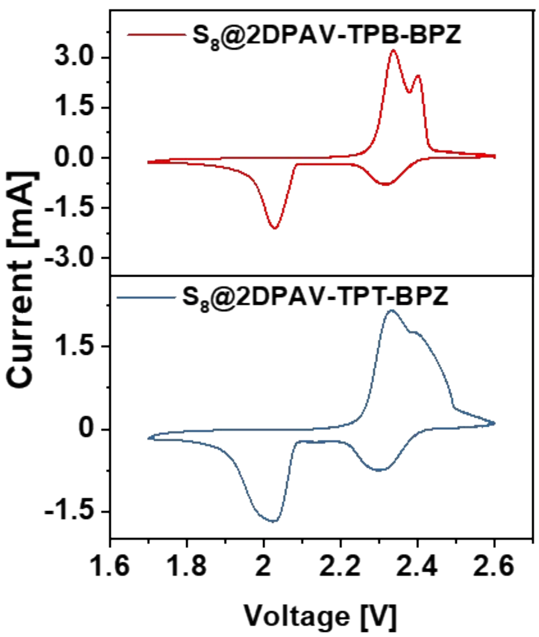

Figure S31: CV curves of the composite electrodes (86% Sulfur) in Li-sulfur batteries.

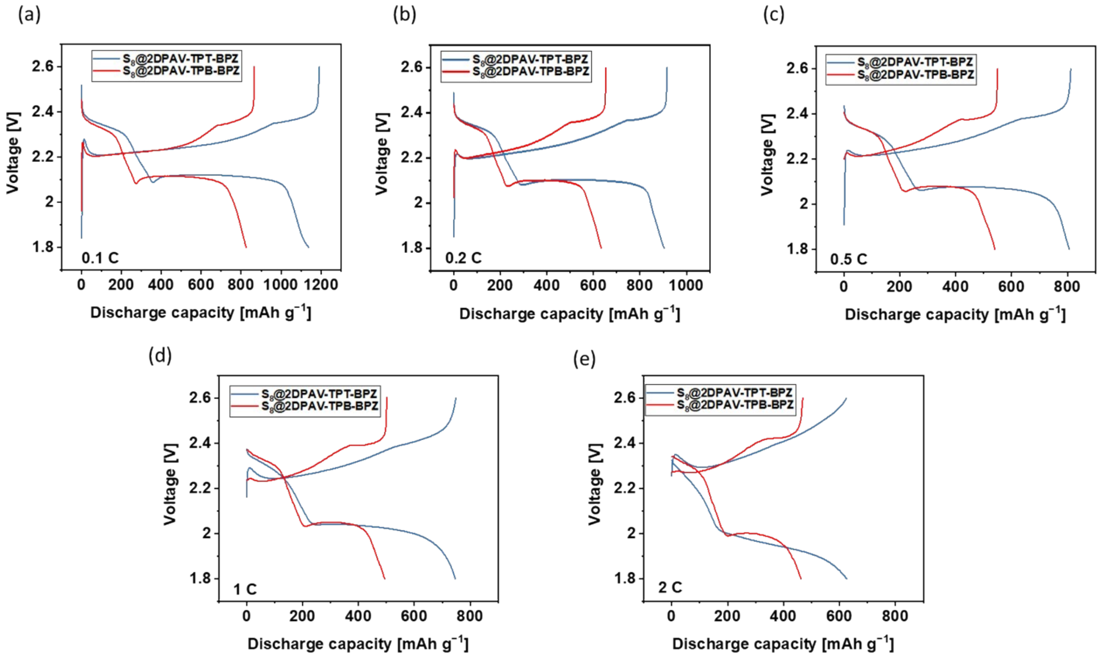

Figure S32: Additional battery data: a)-e) Charge-discharge curves of **S_8@2DPAV-TPT-BPZ** and **S_8@2DPAV-TPB-BPZ** -based batteries under current densities ranging from 0.1-2 C.

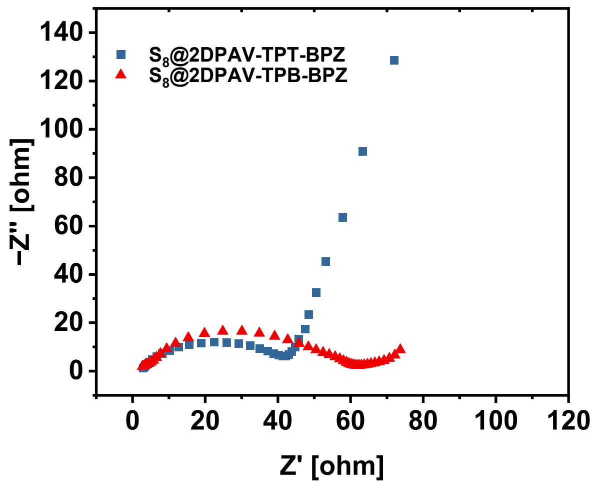

Figure S33: Electrochemical impedance spectra of the batteries containing **S$_8$@2DPAV-TPT-BPZ** and **S$_8$@2DPAV-TPB-BPZ**.

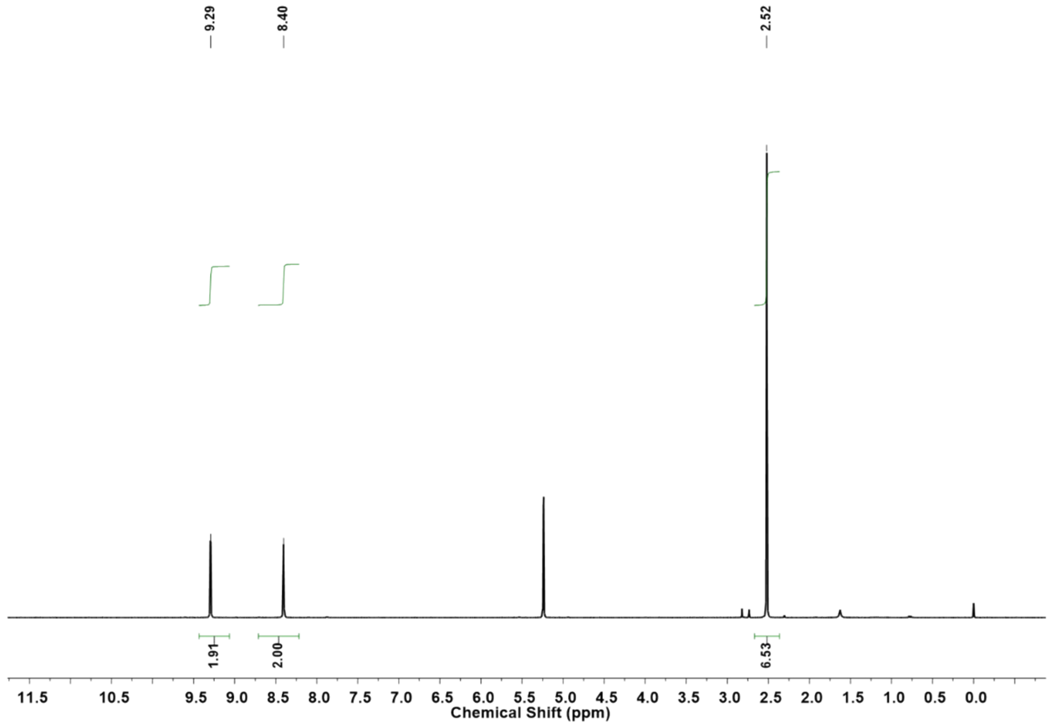

Figure S34: ^{1}H – NMR spectrum of **DMBP** monomer in CD_2Cl_2.

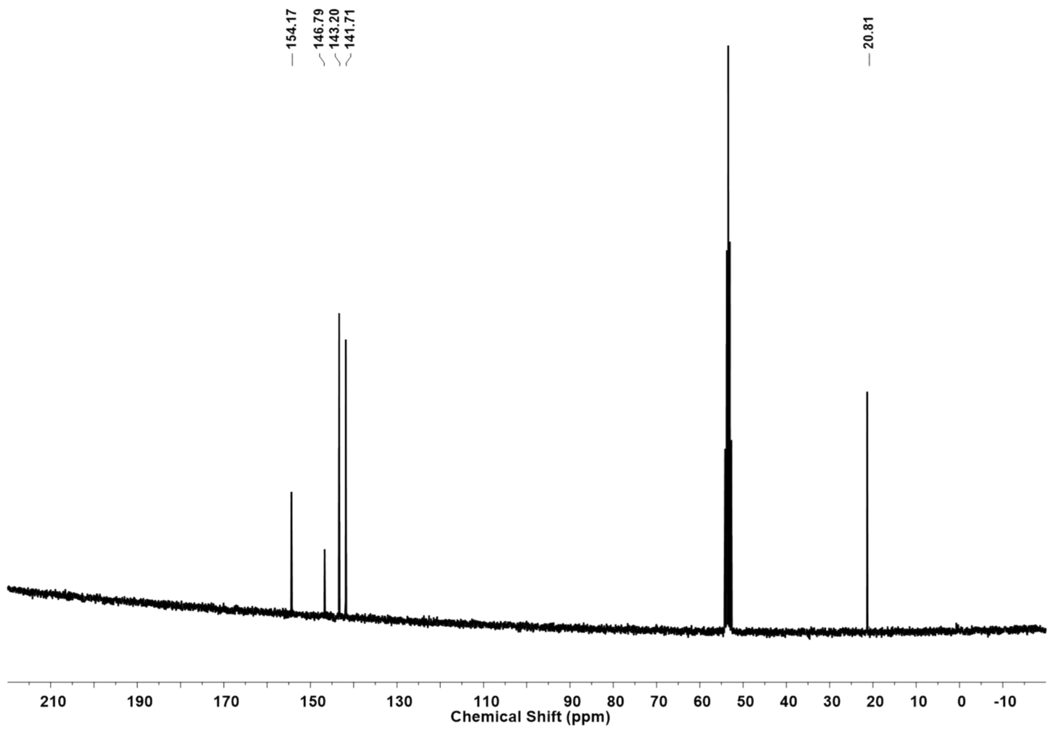

Figure S35: ^{13}C – NMR spectrum of **DMBP** monomer in CD_2Cl_2.

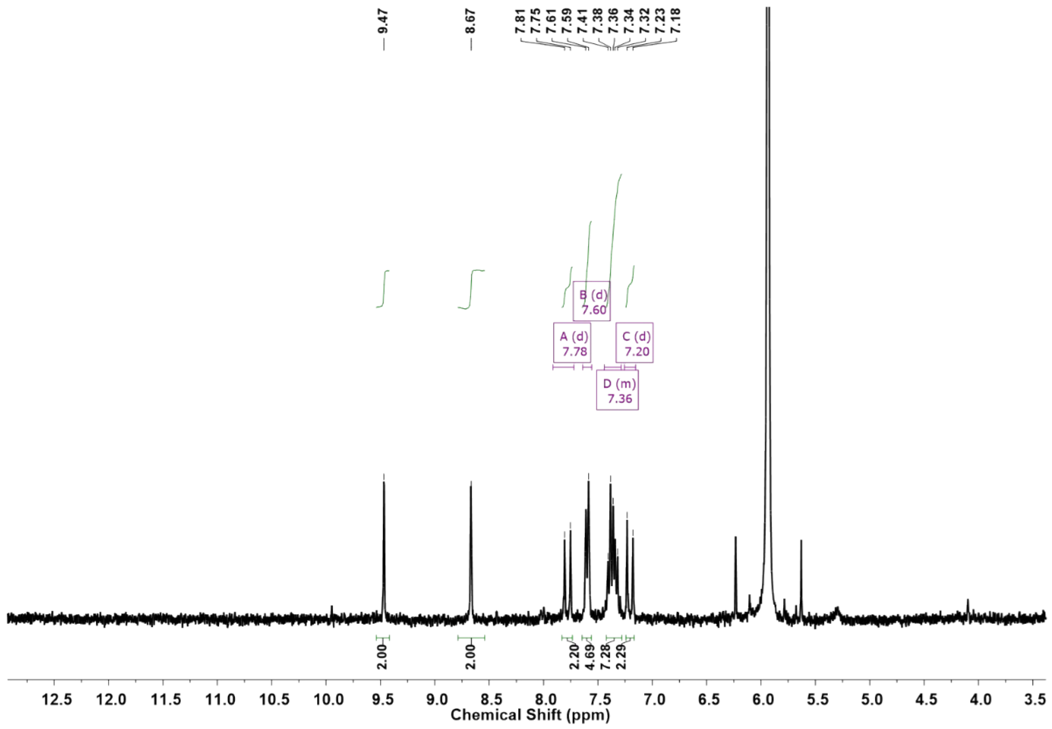

Figure S36: Detailed ^{1}H NMR of **DSBPZ** in $C_2D_2Cl_4$. Coupling constants of vinyl protons clearly show trans configuration.

Section D: Supplementary Tables

Table S1: Screening conditions to obtain crystalline **2DPAV-TPB-BPZ.**

**Entry**	**Conditions**	**T(°C)**	**Product state**	**Crystallinity**
1	30 mg **BAc**	200	Yellow solid	no
2	30 mg **BAc**	180	Yellow solid	no
3	30 mg **BAn**	200	Black carbonized solid	no
4	30 mg **BAn**	180	Yellow, partially carbonized	poor
5	30 mg **BAn**+15 mg **BAc**	200	Yellow, partially carbonized	no
6	30 mg **BAn**+15 mg **BAc**	100	Yellow solid	no
7	30 mg **BAn**+ 15 mg **SBz**	200	Yellow solid	poor
**8**	**30 mg BAn+ 15 mg SBz**	**180**	**Yellow solid**	**high**
9	30 mg **BAn**+ 15 mg **SBz**	150	Yellow solid	moderate

Screening optimization for 2DPAV-TPB-BPZ

Screening conditions based on the synthesis of **2DPAV-TPB-BPZ**, 10 mg **TFPB**, 7,1 mg **DMBP**, 72 h of reaction time. Proportions of benzoic acid (**BAc**), benzoic anhydride (**BAn**) and sodium benzoate (**SBz**) are given in the table. Crystallinity was based of appearance, intensity and peak width of (100) reflex in PXRD.

Table S2: Elemental analysis of 2D PAV-S_8 composites.

Element (% w/w)	S_8@2DPAV-TPB-BPZ	S_8@2DPAV-TPT-BPZ
Sulfur	85.96	85.65

Due to the ultra-low solubility of sulfur in organic solvents, we are unable to remove the excess of sulfur outside of the pore channels of 2D PAVs at such a high sulfur density. Therefore, the gravimetric, thermogravimetric, and elemental analyses show an almost identical sulfur content (80-86 wt%) for the same composite.

Table S3: Literature reported COF-based cathodes for Li-S batteries.

Cathode	Sulfur density [wt%]	Sulfur loading in electrode [mg cm^{-2}]	Capacity @ ca. 0.1 C [mAh g^{-1}]	Capacity @ ca. 2 C & retention [mAh g^{-1}]	Cycling stability: capacity retention [%]	Ref.
**Crystalline sulfurized COF materials**						
**S_8@2DPAV-TPB-BPZ**[a]	86	0.7	794 (0.1 C)	464 (2 C)	73 (0.5 C, 200 cycles)	**This work**
**S_8@2DPAV-TPT-BPZ**[a]	86	0.7	1086 (0.1 C)	628 (2 C)	80.1 (0.5 C, 200 cycles)	
S-DUT-177[b]	62	0.55	720 (100 mA g^{-1})	240 (1 Ag^{-1})	76.6 (500 mAg^{-1}, 500 cycles)	[4]
DUT-185-S_n[b]	60	2.2	620 (100 mA g_{COF}^{-1})	300 (1 Ag^{-1})	73 (250 mA g_{COF}^{-1}, 250 cycles)	[5]
**Amorphous sulfurized COF materials**						
S-COF-1[a]	45	1.0	1179 (0.2 C)	505 (2 C)	45.0 (0.2 C, 140 cycles)	[6]
S-COF-2[b]	43	1.0	1293 (0.2 C)	756 (2 C)	61.9 (0.2 C, 140 cycles)	[6]
cPpy-S-CTF[b]	83	0.8	900 (0.1 C) [c]	600 (2 C)[c]	86.8 (0.5 C, 500 cycles)	[7]
SF-CTF-1[b]	86	0.70	878 (0.1 C)	534 (2 C)	81.6 (1C 300 cycles	[8]
S/CTF-1[b]	62	0.50	670 (0.05 C)	400 (2 C)	85.8 (1 C, 300 cycles)	[9]
FCTF-S[b]	51	1.3	1296 (0.1 C)	500 (5 C)	64 (0.1 C, 150 cycles)	[10]
COF-F-S[b]	62	0.5	962 (0.1 C)	325 (2.0 C)	64.5 (0.1 C 100 cycles)	[11]
S-COF-V[b]	67	-	959 (0.2 C)	431 (6 C)	68.5 (0.2 C, 100 cycles)	[12]
Py-COF/S[a]	70	-	1145 (0.1 C)	659 (5.0 C)	73.8 (5.0 C, 550 cycles)	[13]

[a] Physical sulfurization

[b] Covalent sulfurization

[c] Value was estimated from literature

Table S4: Crystallographic information for **2DPAV-TPT-BPZ**.

Symmetry space group name H-M: 'P-3'

Symmetry cell setting: trigonal

Symmetry Int. Tables number: 147

Lattice parameters

cell length a	40.0467
cell length b	40.0467
cell length c	4.2531
cell angle alpha	90
cell angle beta	90
cell angle gamma	120

Structure parameters in fractional coordinates

**Atom site label**	**Atom type**	**Coord. X**	**Coord. Y**	**Coord. Z**	**U iso**		**Occ.**
C1	C	0.64736	0.29771	0.45312	0	Uiso	1
N2	N	0.31462	0.63213	0.54689	0	Uiso	1
C3	C	0.36638	0.62758	0.54643	0	Uiso	1
C4	C	0.34736	0.59105	0.52284	0	Uiso	1
C5	C	0.36403	0.57142	0.52073	0	Uiso	1
C6	C	0.40048	0.58732	0.54176	0	Uiso	1
C7	C	0.41949	0.62384	0.56885	0	Uiso	1
C8	C	0.40282	0.64349	0.56985	0	Uiso	1
C9	C	0.41574	0.56615	0.53776	0	Uiso	1
C10	C	0.45114	0.57736	0.49003	0	Uiso	1
C11	C	0.46502	0.55516	0.49279	0	Uiso	1

C12	C	0.50086	0.56803	0.45336	0	Uiso	1
N13	N	0.51296	0.5456	0.45821	0	Uiso	1
C14	C	0.49261	0.51076	0.49905	0	Uiso	1
C15	C	0.45672	0.49796	0.53817	0	Uiso	1
N16	N	0.44457	0.52036	0.53367	0	Uiso	1
H17	H	0.32096	0.57809	0.50588	0	Uiso	1
H18	H	0.34901	0.54509	0.50226	0	Uiso	1
H19	H	0.44578	0.6369	0.59097	0	Uiso	1
H20	H	0.4179	0.66978	0.58934	0	Uiso	1
H21	H	0.39947	0.54012	0.5703	0	Uiso	1
H22	H	0.468	0.60306	0.44835	0	Uiso	1
H23	H	0.51794	0.59384	0.42133	0	Uiso	1
H24	H	0.4395	0.47221	0.57017	0	Uiso	1

**Atom label 1**	**Atom-Label 2**	**Bond-distance**	**Bond-symmetry**	**Bond-type**
C1	N2	1.218	6 656	A
C1	N2	1.218	5 556	A
C1	C3	1.268	5 556	S
N2	C1	1.218	5 566	A
N2	C1	1.218	6 556	A
C3	C1	1.268	6 556	S
C3	C4	1.271	.	A
C3	C8	1.271	.	A
C4	C5	1.26	.	A
C4	H17	0.918	.	S
C5	C6	1.27	.	A

C5	H18	0.92	.	S
C6	C7	1.272	.	A
C6	C9	1.269	.	S
C7	C8	1.261	.	A
C7	H19	0.917	.	S
C8	H20	0.919	.	S
C9	C10	1.271	.	D
C9	H21	0.923	.	S
C10	C11	1.263	.	S
C10	H22	0.923	.	S
C11	C12	1.27	.	A
C11	N16	1.225	.	A
C12	N13	1.215	.	A
C12	H23	0.921	.	S
N13	C14	1.227	.	A
C14	C15	1.273	.	A
C14	C14	1.266	4 666	S
C15	N16	1.216	.	A
C15	H24	0.92	.	S

Table S5: Crystallographic information for **2DPAV-TPB-BPZ**.

Symmetry space group name H-M: 'P-3'

Symmetry cell setting: trigonal

Symmetry Int. Tables number: 147

Lattice parameters

cell length a	39.0368
cell length b	39.0368
cell length c	5.42
cell angle alpha	90
cell angle beta	90
cell angle gamma	120

Structure parameters in fractional coordinates

Atom site label	Atom Type	Coord. X	Coord. Y	Coord. Z	U iso		Occ.
C1	C	0.63723	0.29245	1.44953	0	Uiso	1
C2	C	0.30439	0.62648	-0.45672	0	Uiso	1
C3	C	0.35654	0.6089	-0.4022	0	Uiso	1
C4	C	0.3376	0.5808	-0.21164	0	Uiso	1
C5	C	0.35223	0.55631	-0.13287	0	Uiso	1
C6	C	0.38675	0.55943	-0.23897	0	Uiso	1
C7	C	0.40395	0.58519	-0.44262	0	Uiso	1
C8	C	0.38898	0.6095	-0.52333	0	Uiso	1
C9	C	0.40301	0.53819	-0.12644	0	Uiso	1
C10	C	0.44381	0.55176	-0.10891	0	Uiso	1
C11	C	0.45987	0.53559	0.05579	0	Uiso	1
C12	C	0.50103	0.55082	0.07493	0	Uiso	1
N13	N	0.51493	0.53598	0.24957	0	Uiso	1
C14	C	0.49154	0.50665	0.41034	0	Uiso	1
C15	C	0.45027	0.49083	0.3863	0	Uiso	1
N16	N	0.43636	0.50581	0.21304	0	Uiso	1

H17	H	0.3136	0.57863	-0.12095	0	Uiso	1
H18	H	0.33801	0.53708	0.00947	0	Uiso	1
H19	H	0.42784	0.58696	-0.53212	0	Uiso	1
H20	H	0.40252	0.62828	-0.66839	0	Uiso	1
H21	H	0.38394	0.51264	-0.0354	0	Uiso	1
H22	H	0.46303	0.57594	-0.21013	0	Uiso	1
H23	H	0.52062	0.57299	-0.03767	0	Uiso	1
H24	H	0.43046	0.46852	0.49704	0	Uiso	1
H25	H	0.28335	0.59708	-0.45419	0	Uiso	1

**Atom label 1**	**Atom-label 2**	**Bond-distance**	**Bond-symmetry**	**Bond-type**
C1	C2	1.415	6 656	A
C1	C2	1.415	5 556	A
C1	C3	1.417	5 556	S
C2	C1	1.415	5 566	A
C2	C1	1.415	6 556	A
C2	H25	1.024	.	S
C3	C1	1.417	6 556	S
C3	C4	1.416	.	A
C3	C8	1.416	.	A
C4	C5	1.403	.	A
C4	H17	1.024	.	S
C5	C6	1.413	.	A
C5	H18	1.025	.	S
C6	C7	1.416	.	A
C6	C9	1.41	.	S

C7	C8	1.41	.	A
C7	H19	1.023	.	S
C8	H20	1.023	.	S
C9	C10	1.408	.	D
C9	H21	1.025	.	S
C10	C11	1.409	.	S
C10	H22	1.023	.	S
C11	C12	1.411	.	A
C11	N16	1.361	.	A
C12	N13	1.357	.	A
C12	H23	1.022	.	S
N13	C14	1.363	.	A
C14	C15	1.414	.	A
C14	C14	1.412	4 666	S
C15	N16	1.355	.	A
C15	H24	1.021	.	S

Section E: Supplementary References

[1] a) G. Kresse, J. Furthmüller, *Comput. Mater. Sci.,* 1996, ***6***, 15-50; b) Y. Surendranath, M. W. Kanan, D. G. Nocera, *J. Am. Chem. Soc.,* 2010, ***132***, 16501-16509.

[2] J. P. Perdew, K. Burke, M. Ernzerhof, *Phys. Rev. Lett.,* 1996, ***77***, 3865-3868.

[3] S. Grimme, J. Antony, S. Ehrlich, H. Krieg, *J. Chem. Phys.,* 2010, ***132***.

[4] S. Haldar, M. Wang, P. Bhauriyal, A. Hazra, A. H. Khan, V. Bon, M. A. Isaacs, A. De, L. Shupletsov, T. Boenke, J. Grothe, T. Heine, E. Brunner, X. Feng, R. Dong, A. Schneemann, S. Kaskel, *J. Am. Chem. Soc.,* 2022, ***144***, 9101-9112.

[5] S. Haldar, P. Bhauriyal, A. R. Ramuglia, A. H. Khan, S. De Kock, A. Hazra, V. Bon, D. L. Pastoetter, S. Kirchhoff, L. Shupletsov, A. De, M. A. Isaacs, X. Feng, M. Walter, E. Brunner, I. M. Weidinger, T. Heine, A. Schneemann, S. Kaskel, *Adv. Mater.,* 2023, *35*, 2210151.

[6] J.-M. Chen, H. Duan, Y. Kong, B. Tian, G.-H. Ning, D. Li, *Energy Fuels,* 2022, ***36***, 5998-6004.

[7] J. Kim, A. Elabd, S.-Y. Chung, A. Coskun, J. W. Choi, *Chem. Mater.,* 2020, ***32***, 4185-4193.

[8] S. H. Je, H. J. Kim, J. Kim, J. W. Choi, A. Coskun, *Adv. Funct. Mater.,* 2017, ***27***, 1703947.

[9] S. N. Talapaneni, T. H. Hwang, S. H. Je, O. Buyukcakir, J. W. Choi, A. Coskun, *Angew. Chem., Int. Ed.,* 2016, ***55***, 3106-3111.

[10] F. Xu, S. Yang, G. Jiang, Q. Ye, B. Wei, H. Wang, *ACS Appl. Mater. Interfaces,* 2017, ***9*,** 37731-37738.

[11] D.-G. Wang, N. Li, Y. Hu, S. Wan, M. Song, G. Yu, Y. Jin, W. Wei, K. Han, G.-C. Kuang, W. Zhang, *ACS Appl. Mater. Interfaces,* 2018, ***10***, 42233-42240.

[12] Q. Jiang, Y. Li, X. Zhao, P. Xiong, X. Yu, Y. Xu, L. Chen, *J. Mater. Chem. A,* 2018, **6**, 17977-17981.

[13] Y. Meng, G. Lin, H. Ding, H. Liao, C. Wang, *J. Mater. Chem. A,* 2018, **6**, 17186-17191.
